# Side dominance and eye patches obscuring half of the visual field do not affect walking kinematics

**DOI:** 10.1038/s41598-025-90936-x

**Published:** 2025-02-20

**Authors:** János Négyesi, Bálint Kovács, Bálint Petró, Diane Nabil Salman, Ahsan Khandoker, Péter Katona, Mostafa Mohamed Moussa, Tibor Hortobágyi, Kristóf Rácz, Zsófia Pálya, László Grand, Rita Mária Kiss, Ryoichi Nagatomi

**Affiliations:** 1 Department of Kinesiology, Hungarian University of Sports Science, Budapest, Hungary; 2 Neurocognitive Research Center, Nyírő Gyula National Institute of Psychiatry, and Addictology, Budapest, Hungary; 3 CRU Hungary Kft, Budapest, Hungary; 4 Faculty of Sport Science, Ningbo University, Ningbo, China; 5 Faculty of Mechanical Engineering, Department of Mechatronics, Optics and Mechanical Engineering Informatics, Budapest University of Technology and Economics, Budapest, Hungary; 6 Biomedical Engineering Department, Khalifa University, Abu Dhabi, UAE; 7 Department of Neurology, Somogy County Kaposi Mór Teaching Hospital, Kaposvár, 7400 Hungary; 8 Department of Sport Biology, Institute of Sport Sciences and Physical Education, University of Pécs, Pécs, Hungary; 9 Center for Human Movement Sciences, University of Groningen, University Medical Center Groningen, Groningen, The Netherlands; 10 Faculty of Information Technology, Pázmány Péter Catholic University, Budapest, Hungary; 11 Designing Future Health Initiative (DFHI), Promotion Office of Strategic Innovation, Tohoku University, Sendai, Japan

**Keywords:** Electromyography, Gait, laterality, Motion capture, Vision, Motor control, Biomedical engineering

## Abstract

Vision plays a fundamental role in the control of human locomotion, including walking gait. Given that side-dominance is associated with differences in motor control, the present study aimed to determine if patches obscuring half of the visual field affect left- and right-side dominant individuals’ gait kinematics and accompanying leg muscle activation differently. Healthy right- (*n* = 15, age = 28.2 ± 5.5 years) and left-side (*n* = 9, age = 27.9 ± 5.8 years) dominant participants performed 10 min of walking trials on a treadmill at a self-selected speed with 5 min of rest between three randomized trials, i.e., wearing clear glasses or glasses with left-or right half-field eye patching. In addition to a set of spatiotemporal and kinematic gait parameters, the average activity during the separated gait cycle phases, and the start and end of muscle activation in % of the gait cycle were calculated from five muscles in three muscle groups. Our results indicate that gait kinematics of left- and right-side dominant participants were similar both in their dominant and non-dominant legs, regardless of half-field eye patching condition. On the other hand, inter-group differences were found in selected kinematic variables. For instance, in addition to larger but less variable step width, our results suggest larger ankle and knee ROM in right- vs. left-sided participants. Furthermore, medial gastrocnemius and biceps femoris muscle activation showed selected differences at certain phases of the gait cycle between participants’ dominant and non-dominant legs. However, it was also unaffected by the half-field eye patching condition. Moreover, the endpoint of medial gastrocnemius activation was affected by side-dominance, i.e., its activation ended earlier in the non-dominant leg of right- as compared to left-side dominant participants. Our results suggest no major differences in walking gait kinematics and accompanying muscle activation between half-field eye patching conditions in healthy adults; nevertheless, side-dominance may affect biomechanical and neuromuscular control strategies during walking gait.

**Supplementary Information:**

The online version contains supplementary material available at 10.1038/s41598-025-90936-x.

## Introduction

Vision plays a fundamental role in the control of human locomotion^1^. To illustrate, deficits in peripheral vision can cause a loss of balance, induce a cautious gait and may require probing the ground with the foot^2–4^. Adaptations to deficits in peripheral vision emerge and anticipatory actions are generated through feedforward mechanisms. Humans show a population-level bias toward using one side of the brain for functions such as language, speech, or face recognition. This phenomenon is called hemispheric lateralization. Specifically, the evolutionary specialization of the left hemisphere for skilled motor activities^5–7^induced the tendency toward right-handedness that has been present throughout human history, regardless of cultures and continents^8–10^. For example, as a result of hemispheric lateralization, our previous study suggested that left vs. right-side dominant healthy individuals perform even such simple tasks as a unilateral stance with different biomechanical and neuromuscular control strategies^11^.

With respect to vision, healthy adults do not perceive the left and right sides of space equally, showing a leftward spatial bias on visuospatial tasks^12,13^. Theoretically, with the use of half-field eye patching glasses, a lack of visual input from one hemifield may decrease the input to the contralateral cortical and sub-cortical structures. Therefore, if the leftward bias results from right-hemisphere dominance on visuospatial tasks, blocking left visual input may affect the right hemisphere, i.e., moving the bias rightward^14,15^. Nevertheless, considering that previous studies used half-field eye patches only in patients with spatial neglect^16–18^but not in healthy adults, it remains unknown whether left or right half-field eye patching would be effective in modifying healthy individuals’ spatial bias when they perform visuomotor tasks or even while walking considering that visuomotor coordination has a crucial role in locomotion^19,20^.

Proprioception is the sensation of the static and dynamic position and motion of limbs, an ability that also contributes to joint stability^21–24^. Although joint position sense (*JPS*) error is the most widely used objective measure of proprioceptive function at the knee^25^and ankle^26^joints, active or passive movement training, somatosensory stimulation training, force reproduction, and somatosensory discrimination training are also types of proprioceptive interventions^27^. Feedback from lower extremity proprioceptors shapes postural stability in standing^28^and gait^29^. Gait analysis can identify asymmetries and compensations in locomotion. Such analyses can include kinematics using motion capture (MoCap) and neural control via electromyography (EMG). MoCap data informs us about center of mass (CoM) displacement and other descriptive aspects of walking balance stability. Analyses of EMG data recorded from the three cardinal muscle groups (anterior leg muscles, posterior leg muscles, trunk muscles) can provide insights into the neural control of posture^30^and the modular organization and neural control of walking balance^31–33^. In healthy participants, vision regulates many gait-related parameters including walking speed, cadence, stride length or even gait direction^34,35^. Our understanding of the inter-hemispheric differences regarding their role in gait is from studies with hemiparetic stroke patients^36,37^. It was shown that while the right hemisphere is mainly involved e.g., in spatial orientation and the position control, the left hemisphere is related to the motor control, trajectory, and movement dynamics. A previous study^38^aimed to examine the cortical control of gait in healthy humans using functional magnetic resonance imaging (fMRI) and found extensive activations of visual-related areas during walking vs. standing but no inter-hemispheric differences in blood oxygenation level dependent (BOLD) activation. The effects of half-field left or right eye patches on muscle activation pattern have not been well studied. A half-field left or right eye patch affects brain activation in the opposite hemisphere^39^ which is involved in gait control. Because presumably a patch would affect not only structures involved in visual processing, but may be this idea then can be extended to motor areas so that the neural drive to the muscles is also affected.

Walking requires the integration of visual flow, dynamic balance, and a multitude of sensory inputs^40^. Left- and right-handers show differences not only in cerebral cortical anatomy^41^but also in biomechanical and neuromuscular stance control strategies^11^and visual recognition^42^. It is, therefore, also possible that patches obscuring half of the visual field may affect left and right-handed individuals’ locomotion differently. To the best of our knowledge, there is no data in the literature on the interaction between vision, gait parameters, and side-dominance. Because side-dominance is associated with differences in brain morphology as well as in anatomical and functional lateralization, we hypothesized that patches obscuring half of the visual field affect left and right-side-dominant individuals’ gait kinematics and leg muscle activation differently. Specifically, given that left-handed subjects had fewer asymmetries in the topological organization of their human cortical anatomical network^43^, we hypothesized that left-side dominant participants’ walking gait kinematics and muscle activation would not differ during left and right half-field eye patching between their dominant and non-dominant sides. However, because of a paucity of data concerning hemispheric specialization of the neural control of leg muscles during walking with right hemispheric lateralization of knee joint proprioception and single leg balance tasks^44–47^, we tentatively hypothesize the presence of right hemispheric lateralization during treadmill walking. To test this hypothesis, we explored the differences in gait parameters between left- and right-side dominant healthy adults in three conditions, i.e., walking on a treadmill at a self-selected pace with (i) clear glasses, (ii) glasses with right half-field eye patching, or (iii) glasses with left half-field eye patching. Because postural control during upright standing involves the activation of three muscle groups^30^, we also aim to supplement our kinematic data with EMG data analyses of the cardinal muscles that could help better understand how the CNS sends inputs to the muscles to control gait^48^. Our study proposal fits under the current efforts to understand the underlying working mechanisms of laterality.

## Materials and methods

### Participants

Sample size calculations (G*Power 3.1.7^49^) based on a previous study^48^ that aimed to determine the effects of the influence of visual information on multi-muscle control during quiet stance, revealed that a total sample size of 16 would be appropriate for detecting significant differences between the groups, assuming a type I error of 0.05 and power of 0.80.

Considering potential drop-outs, we recruited 9 left-side dominant (age = 27.9 ± 5.8 years; height = 179.4 ± 8.1 m; mass = 75.3 ± 8.1 kg; 3 female) and 15 right-side dominant (age = 28.2 ± 5.5 years; height = 173 ± 8.2 m; mass = 66.6 ± 15.1 kg; 6 female) participants with no reported neurological deficits or sensorimotor impairment. Participant recruitment and data collection was scheduled from December 1st, 2020 to February 30th, 2021. Handedness was determined using the Edinburgh Handedness Inventory^50^, a scale that is used to measure the degree of hand laterality in daily activities such as writing, drawing, throwing, brushing teeth, opening a box, striking a match and using a pair of scissors, knife, spoon, and a broom. Leg dominance was determined by one- or two-foot item skill tests such as kicking a ball or stepping up on a chair^51^. The laterality index for both handedness and footedness was calculated by summing the number of tasks performed with the right limb and the number of tasks performed with the left limb (L) as follows: (R - L) / (R + L). Laterality index for both handedness and footedness were 0.9 ± 0.1 in right-side dominant and − 0.9 ± 0.1 in left-side dominant participants, showing strong right- or left-side dominance, respectively. Both verbal and written explanations of the experimental protocol were given in accordance with the declaration of Helsinki. The study was carried out following the recommendations of the University Ethical Committee of the Hungarian University of Sports Science (Approval No. TE-KEB:3:2021). Informed consent was obtained from all subjects and/or their legal guardian(s).

### Experimental design and procedures

**Fig. 1:**
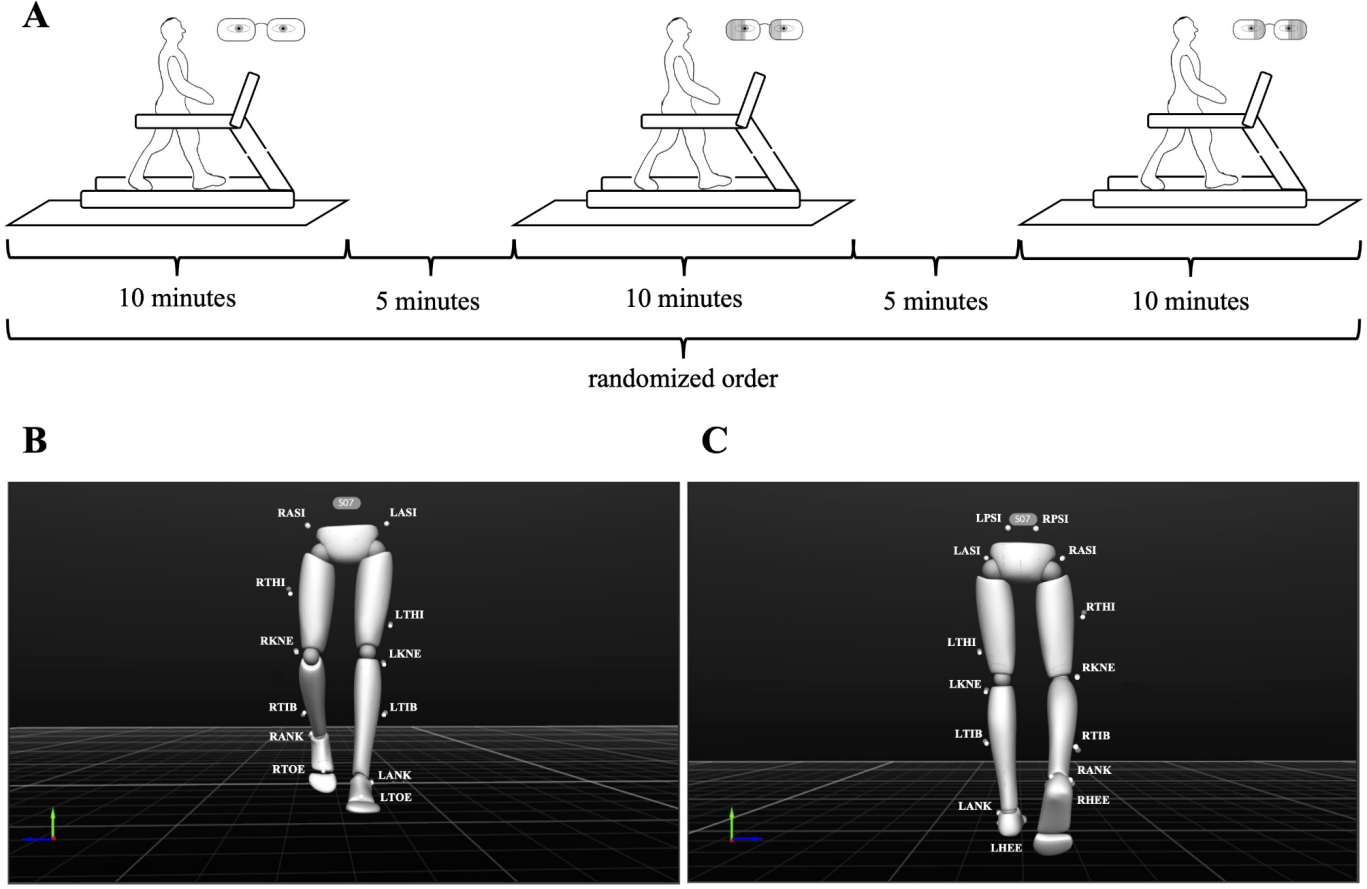
Experimental setup. *Panel A*: Schematic illustration of the experimental setup. *Panel B and C*: 3D avatar of a representative subject’s lower limbs and the placement of the reflective markers for motion capture measurements during walking from the front and back sides, respectively. *Markers*: LANK/RANK: left/right lateral malleolus; LASI/RASI: left/right anterior superior iliac spine; LHEE/RHEE: left/right heel (bisection of the distal aspect of the posterior calcaneum); LKNE/RKNE: left/right knee (lateral epicondyle of the femur); LPSI/RPSI: left/right posterior superior iliac spine; LTHI/RTHI: left/right thigh (not an exact location, only to aid with sides); LTIB/RTIB: left/right shank (not an exact location, only to aid with sides); LTOE/RTOE: left/right toes (between the distal ends of the 1st and 2nd metatarsi).

Considering that walking on a motorized treadmill can be an unfamiliar experience for some individuals, each measurement was preceded by a 6-minute familiarization time^52,53^ at a self-selected walking speed. Participants were allowed to grasp the handrail while walking on the treadmill only during the familiarization period if it was needed. Then, participants performed 10 min of walking trials in 3 different visual conditions, i.e., walking with (1) clear glasses (CLR), (2) glasses with right half-field eye patching (RFP), or (3) glasses with left half-field eye patching (LFP) in a randomized order (Fig. 1A). Gait analysis measurements were performed by a researcher (KR) with the requisite familiarity with gait analysis. Participants spent their rest periods with no glasses on and only put them on immediately before starting the familiarization to rule out any adaptation period that could have affected the results. Participants were asked not to watch their motions while walking but to look ahead and avoid extraneous movements. Participants received 5 min of rest between each condition.

To evaluate the effects of half-field eye patching and side-dominance on left- and right-side dominant participants’ walking gait kinematics, an optical motion capture system (OptiTrack, 18 pieces of Flex13 cameras, NaturalPoint Inc., Oregon, USA) was used. We placed 16 retro-reflective markers (1/2” diameter) on both lower limbs following the ‘conventional lower body’ model provided by Motive capture software. The three-dimensional marker trajectories were recorded with a sampling frequency of 100Hz using OptiTrack’s Motive: body software (version 2.3). It was previously examined^54^ that the accuracy of the OptiTrack optical motion capture system is more than sufficient for measuring full-body human kinematics with skin-mounted markers in a large capture volume. The front and back sides of the 3D avatar of a representative participant during walking are shown in Fig. 1B and C, respectively.

In addition, we recorded EMG signals using a Cometa Wave Plus wireless EMG system with Mini Wave Waterproof units and the EMGandMotionTools 7.0.10.0 software (Cometa S.r.l., Bareggio MI, Italy). EMG signals were recorded on both sides of the body using surface bipolar electrodes. No additional skin preparation was performed before electrode placement unless the EMG system reported insufficient conductivity, in which case the area was shaved and cleaned with alcohol. The measured muscles and muscle groups were the following: (1) posterior muscles: medial gastrocnemius and biceps femoris, (2) anterior muscles: tibialis anterior and rectus femoris, (3) rectus abdominis. Electrodes (Blue Sensor M-00-S/25, Ambu, Denmark) were placed according to the recommendations of the SENIAM project (seniam.org) on the muscular belly with an inter-electrode distance of approximately 20 mm. The sampling frequency was 2000 Hz. The EMG phases were determined by the synchronized joint angle data.

### Data analyses

#### Kinematic data acquired from MoCap analysis

Walking trials are usually broken up into gait cycles consisting of stance and swing phases delimited by instances at which heel strike (HS) and toe-off (TO) events occur, respectively. For this analysis, the optical motion capture data was decomposed into left and right gait cycles where a left gait cycle was defined as the period from one left HS to the next left HS and a right gait cycle as the period from one right HS to the next right HS. Motion capture marker trajectories were filtered using a 4th -order Butterworth low pass filter with a cut-off frequency of 6 Hz^55^and decomposed into gait cycles based on the Zeni formula^56^, according to which they take place at the local extremities (HS-maxima, TO-minima) of the anteroposterior component of the hip-heel distance of the respective sides, based on the hip (LASI/RASI) and heel (LHEE/RHEE) markers. Phase I represents the braking phase (from initial ground contact to mid-stance), phase II is the push-off phase (from mid-stance to take off), and phase IV starts at 10% of the gait cycle until the ground contact itself, which represents the pre-activation of each leg. A static standing pose was recorded before recording marker displacements during walking to optimize the model to the participant’s body dimensions.

To compare gait cycles and evaluate gait patterns for the different participants and conditions, spatiotemporal and kinematic gait parameters were computed and analyzed for both left and right gait cycles. Spatiotemporal parameters were determined both for the dominant and for the non-dominant sides using MATLAB^®^(MATLAB R2021b, MathWorks, Natick, MA, USA) and included stride time, length and speed, step time, length and speed, and the cadence. Minimum toe clearance (MTC) and foot velocity at MTC (velMTC) were also computed as they have been reported as important parameters in describing stability and gait control mechanisms^57,58^. For each of these spatiotemporal parameters, descriptive statistics variables (i.e., mean, median, mode, standard deviation, minimum, maximum, skewness, kurtosis, root mean square value [RMS]) were computed to assess and compare the variability in gait parameters across each walking trial. Moreover, detrended fluctuation analysis (DFA) was used to determine long-range correlations in stride as well as step time, length and speed, MTC and velMTC time series. The window size used for the DFA was 4 to N/4 for the overall alpha, N/52 to N/20 for alpha1, and N/8 to N/4 for alpha2, where N is the total number of strides in the recording^59–61^.

To obtain joint kinematics, hip, knee, and ankle joint flexion-extension was calculated for each stride using three-dimensional inverse kinematics in OpenSim 4.3 (SimTK v. 4.0.1.)^62,63^. Using MATLAB^®^, the average joint angle expressed as a function of the gait cycle percentage was computed as the mean of the joint angle across all recorded gait cycles at every point along the time-normalized gait cycle. A set of variables was then determined for each averaged joint angle cycle such as the range of motion, peak flexion, peak extension, initial contact flexion and midstance minimum flexion. The average standard deviation of joint angles across different gait cycles of the same trial was also assessed in the same way as the average joint angle was^64^. Finally, the lateral pelvic translation motion was analyzed using MATLAB^®^ to check for lateral deviation of participants during the different trials of walking experiments. The distribution of the average lateral pelvic translation across gait cycles (avg_pelvis_tz) was computed and assessed by its trend and central tendency variables.

In contrast, the change in the interquartile range (IQR) of the pelvic translation between gait cycles (iqr_pelvis_tz) was used to quantify the number of corrective cycles defined by cycles for which the IQR is below the 2nd percentile or above the 98th percentile. Butterfly plots representing the average pelvic movement during a gait cycle were also generated by plotting the average anterior-posterior pelvic movement against the lateral pelvic movement with correction for displacement in the line of progression^65^. Those butterfly plots were used to calculate the area under the curve for each side (left and right) as well as the pelvic lateral displacement (PLD) defined as the peak-to-peak displacement of the pelvis in the medial-lateral direction during a gait cycle. All gait parameters and motion capture analysis variables used in this study are summarized and defined in Supplementary Table 1.

#### EMG data

The EMG data from all 10 channels was obtained from five different muscles of the left and right lower limbs during each standing condition. The pre-processing of the EMG signal was done in line with the methods used in our previous study^11^. Briefly, to remove any signal noise, custom wavelet filters (30–500 Hz) were applied using symlet mother wavelet (Sym5)^66^ in MATLAB^®^^67^. The filtered EMG signals were then smoothed using root mean square (RMS) with a 100ms window size. The RMS EMG signals were normalized to the peak activity during clear-eyed walking for each muscle. Then the average activity during the separated gait cycle phases (I, II, and IV) was calculated.

Furthermore, the start and end of muscle activation were measured in % of the gait cycle. The onset and offset of the muscles were determined using the 5SD method. A 500ms interval was averaged during the standing period, and SD was also calculated for each muscle. When the EMG signal became higher than the 5SD of this reference period, the muscle was considered active, and when the EMG signal dropped below the 5SD, the muscle was considered deactivated^68^.

### Statistical analyses

We report the data as mean ± SD. Variables were normally distributed, measured by Shapiro–Wilk’s test of normality and visual inspection of their histograms. The analyses were done using the SPSS Statistics Package (version 28.0.1, SPSS Inc., Chicago, IL). Separate multivariate analysis of variance (MANOVA) was applied to comprehensively assess the interaction and/or main effect of kinematic gait parameters for the Group (side dominance: right-side dominant, left-side dominant) during gait. In case of significant main effect or interaction, a series of Group × Glass (CLR, RFP, LFP) × Leg (dominant, non-dominant) mixed ANOVA and planned post-hoc tests with Bonferroni correction for multiple comparisons were performed to statistically investigate the effects of half-field eye patching and side-dominance on kinematic data. Regarding EMG variables (RMS of each gait phase, start and end of muscle activation) of each muscle (medial gastrocnemius, biceps femoris, tibialis anterior, rectus femoris, rectus abdominis), separate Group × Glass × Leg mixed ANOVAs with repeated-measures on Glass and Leg were performed to statistically investigate the effects of half-field eye patching and side-dominance on muscle activation. Compound symmetry was evaluated with Mauchly’s test, and the Greenhouse-Geisser correction was used when data violated the assumption of sphericity so that when the Epsilon was less than 0.75 for Mauchly’s test of sphericity, we used the Greenhouse–Geisser-corrected value and the Huynh–Feldt- corrected value for epsilon greater than 0.75. Complementary post hoc analyses (independent-sample t-tests for Group main effect, paired-sample t-tests for Glass or Leg main effect) were used when indicated. Cohen’s effect size, d, was also computed as appropriate. Additionally, effect sizes of repetition factors will be expressed using partial eta squared (η_p_^2^)^69^. Statistical significance was set at *p* < 0.05.

## Results

**Fig. 2:**
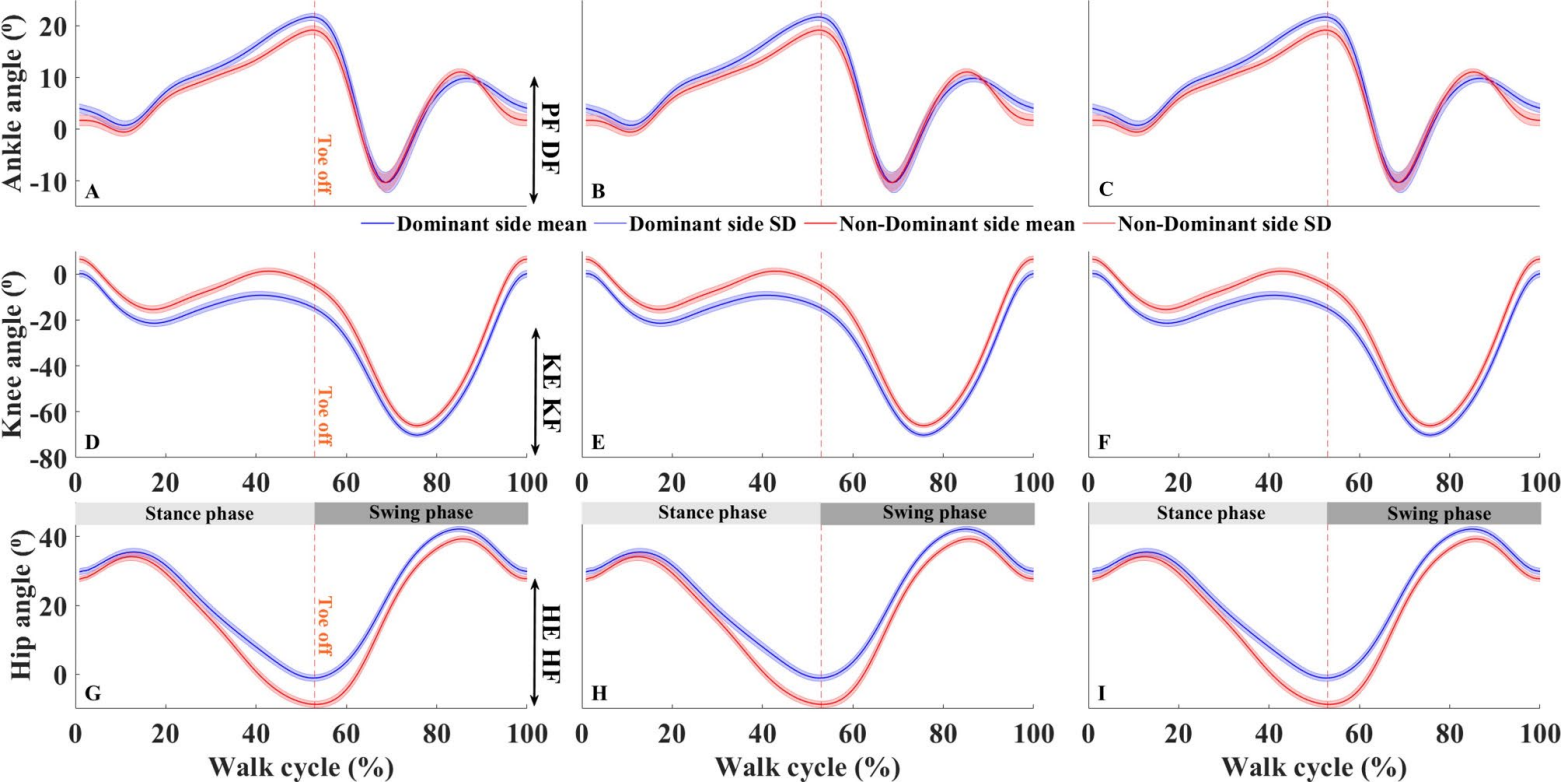
**Mean and SD joint angle displacement of the ankle**,** knee**,** and hip joints during different walking conditions in a representative participant**,** for both the dominant and non-dominant legs.** Each row represents a different joint: the first row (Subplots A–C) corresponds to the ankle joint, the second row (Subplots D–F) to the knee joint, and the third row (Subplots G–I) to the hip joint. Each column represents different walking conditions: (i) Subplots A, D, and G: show joint displacement during normal vision; (ii) Subplots B, E, and H show joint displacement during left half-field eye patching condition; and (iii) Subplots C, F, and I show joint displacement during the right half-field eye patching condition. The color-matched shaded area is the corresponding SD. Orange vertical dashed lines represent the toe-off instant. PF: plantarflexion, DF: dorsalflexion, KE: knee extension, KF: knee flexion, HE: hip extension, HF: hip flexion.

All MoCap results can be found in Supplementary Tables 2–16. Figure 2 shows the mean and SD joint angle displacement of the ankle, knee, and hip joints during different walking conditions in a representative participant, for both the dominant and non-dominant legs. Multivariate ANOVA (MANOVA) demonstrated that there were no significant Glass main effects or interactions with Group and/or Leg (all *p* > 0.05) on any kinematic gait parameters suggesting that gait kinematics of left- and right-side dominant participants were similar both in their dominant and non-dominant legs, regardless of half-field eye patching condition. However, we found Group main effects on selected gait kinematic variables. Specifically, MANOVA revealed Group main effects in stride time (F_13,120_ = 2.460, *p* = 0.005, η_p_^2^ = 0.210), stride speed (F_12,121_ = 2.876, *p* = 0.002, η_p_^2^ = 0.222), step time (F_13,120_ = 1.990, *p* = 0.027, η_p_^2^ = 0.177), and MTC (F_13,120_ = 2.345, *p* = 0.008, η_p_^2^ = 0.203) with the post-hoc analyses showing smaller DFA overall/1/2 values in right- vs. left-side dominant participants (all *p* < 0.05) suggesting that long-range correlations in MTC time series exist irrespective of half-field eye patching condition. In addition, MTC SD (*p* < 0.001, d = 0.590), IQR (*p* = 0.002, d = 0.545), and skewness (*p* = 0.026, d = 385) were larger in right- vs. left-sided participants. Moreover, we found significant Group main effect (F_13,120_ = 4.169, *p* < 0.001, η_p_^2^ = 0.311) with Group × Leg interaction effect (F_13,120_ = 1.979, *p* = 0.028, η_p_^2^ = 0.177) in step width with the post-hoc analyses showing larger mean (*p* = 0.040, d = 0.365), min (*p* < 0.001, d = 0.842), median (*p* = 0.046, d = 0.354), mode (*p* < 0.001, d = 0.842), Q1 (*p* = 0.011, d = 0.454) and IQR (*p* = 0.027, d = 0.414) values but smaller SD values (*p* = 0.010, d = 0.483) suggesting larger but less variable step width in right- vs. left-sided participants (Supplementary Table 7). Furthermore, MANOVA detected Group main effect (F_4,129_ = 4.329, *p* = 0.003, η_p_^2^ = 0.118) and Group × Leg interaction effect (F_4,129_ = 5.931, *p* < 0.001, η_p_^2^ = 0.155) in ankle joint kinematics with the post-hoc analyses showing larger ROM (*p* = 0.011, d = 0.446) and initial contact flexion (*p* = 0.003, d = 0.565) values but smaller peak extension (*p* = 0.003, d = 0.508) values in right- vs. left-side dominant participants (Supplementary Table 13). Knee angle ROM was also larger in right- vs. left-side dominant participants (MANOVA Group main effect: F_5,128_ = 4.905, *p* < 0.001, η_p_^2^ = 0.161) (Supplementary Table 13). Right-side dominant participants had larger mode value for avg_pelvis_tz (*p* = 0.009, d = 0.635) with smaller SD (*p* = 0.001, d = 0.808) as compared to left-side dominant participants suggesting larger but less variable average lateral pelvic translation during gait (Supplementary Table 13).

**Fig. 3:**
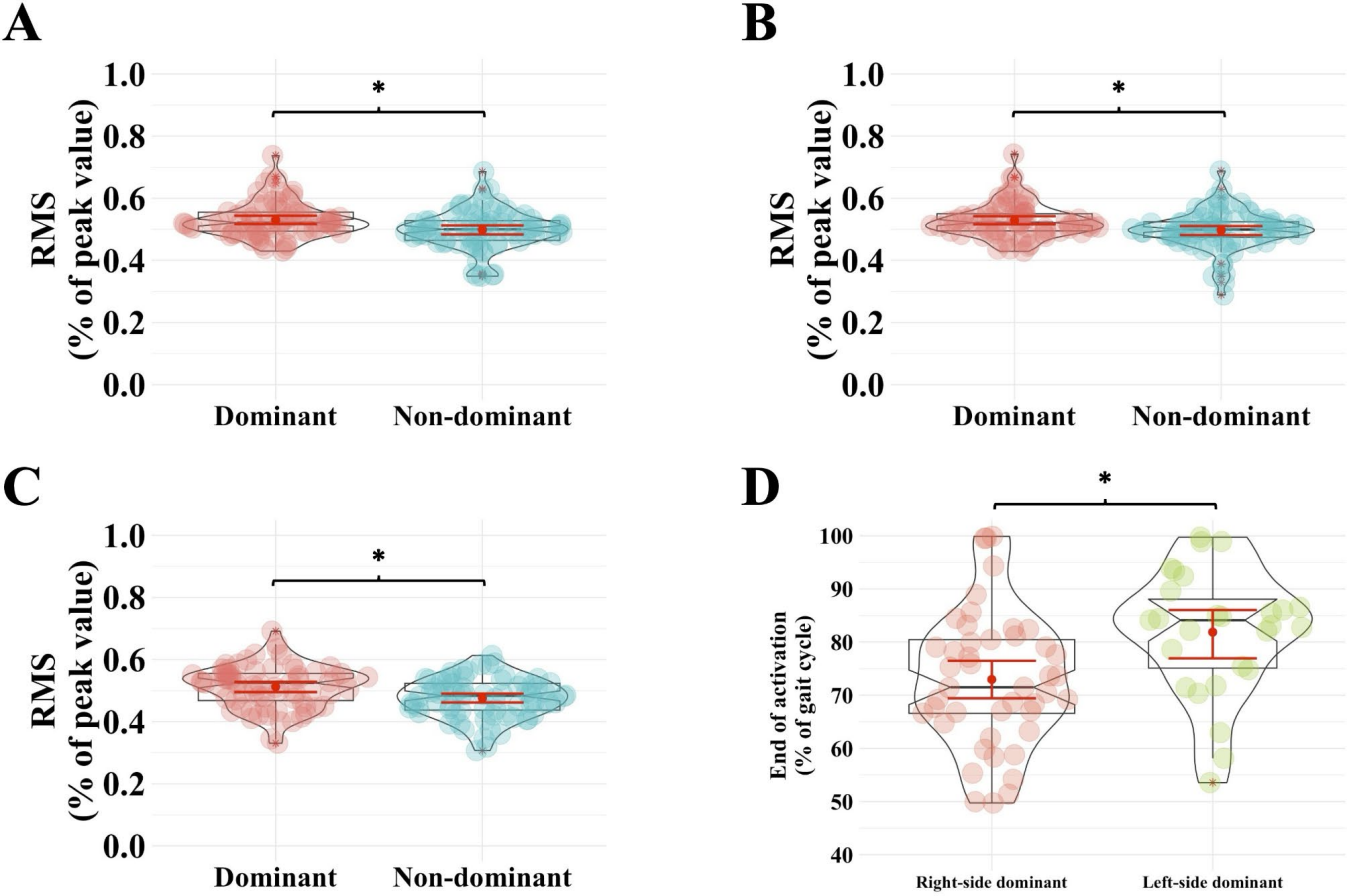
EMG results. Participants had lower medial gastrocnemius RMS values in their non-dominant vs. dominant leg during phase II (*Panel A*) and IV (*Panel B*), regardless of group and eye patching condition. Participants had lower biceps femoris RMS values in their non-dominant vs. dominant leg during phase II (*Panel C*), regardless of group and eye patching condition. *Panel D*: Medial gastrocnemius activation ended earlier in the non-dominant leg of right- as compared to left-side dominant participants. The violin plots (transparent color) over the boxplots represent the data distribution. The boxplots show the median, the upper, and lower quartiles, and the min and max values. Red error bars within the boxplots represent the 95% confidence interval (CI) around the mean (red dot). Each data point is an individual token: the horizontal jitter is not meaningful and is only used for visualization purposes. * indicates differences between the left and right arms in both standing conditions (*p*< 0.05). The graphs were created using RStudio software (version 2023.12.0 + 369)^79^.

Regarding EMG data, mixed ANOVA revealed significant leg main effects for medial gastrocnemius RMS during phase II (F_1,22_ = 4.612, *p* = 0.043, _p_^2^ = 0.173) (Fig. 3A) and IV (F_1,22_ = 4.476, *p* = 0.046, _p_^2^ = 0.169) (Fig. 3B) and for biceps femoris during phase II (F_1,22_ = 4.869, *p* = 0.038, _p_^2^ = 0.181) (Fig. 3C) with the post-hoc analyses showing lower muscle activation in their non-dominant vs. dominant leg. There was only one significant Group × Leg interaction effect in medial gastrocnemius regarding the end of its activation. Post-hoc analysis revealed that the activation ended earlier in the non-dominant leg of right- as compared to left-side dominant participants (*p* = 0.005, d = 0.072) (Fig. 3D).

## Discussion

We present the results focusing on the comparisons related to the hypotheses. Accordingly, Hypothesis 1 examined if patches obscuring half of the visual field would differently affect gait kinematics and accompanying leg muscle activation. Our results indicate that the half-field eye patching condition did not affect participants’ gait kinematics, regardless of leg and group. The secondary hypotheses of dominant vs. non-dominant limbs and left vs. right-side dominant people seem to have been partially supported given that differences were found between left- and right-side dominant participants’ selected kinematic variables. Medial gastrocnemius and biceps femoris muscle activation showed selected differences at certain phases of the gait cycle between participants’ dominant and non-dominant legs, however, it was also unaffected by the half-field eye patching condition. Moreover, the endpoint of medial gastrocnemius activation was affected by side-dominance, i.e., its activation ended earlier in the non-dominant leg of right- as compared to left-side dominant participants, regardless of half-field eye patching condition. Overall, we observed no major differences in walking gait kinematics and mean EMG activity of any muscle between half-field eye patching conditions. Although, the timing of muscle offset was slightly different between left and right-side dominant participants’ medial gastrocnemius muscle, its practical significance in the dexterity context is questionable.

### Kinematic data

Against the hypothesis, we observed no effects of half-field left or right eye patches on gait kinematics. Eye patches were previously used to facilitate vision only in patients with spatial neglect^16–18^. A half-field left or right eye patch interferes with visual input to the structures in the opposite hemisphere which are known to be involved in gait control^39^. We thus argued for the first time that wearing a half-field left or right eye patch affect gait kinematics even in healthy adults. At the extreme, humans are unable to maintain a straight path of walking with vision blocked and veer^70^, assigning a fundamental role to vision in locomotion^19,20^. One reason we failed to observe an effect of eye patches on gait kinematics was that we tested these effects while our healthy volunteers walked on a treadmill. Walking on a treadmill limits the degrees of freedom of how and where the foot can be placed on the belt^52,71,72^. The spatial and temporal restrictions by the narrow and moving belt and the handrails induce a gait that is reactive to the environment. Gait is thus not self-generated, minimizing the potential for subtle kinematic adjustments to manifest in response to the perturbations caused by half-field eye patches. Treadmill walking may be associated with joint kinematics different from overground walking; by enforcing a constant speed and limited width, one could argue that performing micro compensations to keep balance (e.g., slowing down temporarily, or veering off to the side) that enforces larger compensatory behavior (e.g., change in joint angle, stumbling) may potentially affect the required kinematic adjustments. Had we asked our participants to walk in a hallway, we might have been able to discern the effects of the patch-induced perturbations on gait kinematics. Nevertheless, our results are align with a previous study that demonstrated that lateralized vision loss alone is not the primary cause of walking difficulties^73^. A second reason could be that the half-field eye patches under the current experimental conditions and sample examined, produced minimal perturbations relative to the signal represented by gait kinematics. This is consistent with the idea that visual perturbations as great as vision block might be needed to modify healthy individuals’ gait^70^and even then proprioceptive training can reduce the resulting perturbations in gait^74^. A third reason could be related to our choice of measuring laterality. While handedness is characterized by specific cerebral anatomy^41^and affects even biomechanical and neuromuscular control strategy^11^and visual recognition^42^, defining side-dominance based on handedness and footedness in relation to gait was not an optimal choice. A future study with a similar aim should define side-dominance based on eye-dominance and examine the effects of half-field left or right eye patches on gait kinematics while walking freely instead of on a treadmill.

Furthermore, in a previous study^59^, long-range correlations (by DFA exponents) between the stride-to-stride MTC variables were investigated to understand the aging effect on gait control mechanisms to maintain balance. The breakdown of long-range correlations in MTC time series signals has been linked with the degeneration of the underlying levels of input (external, such as audiovisual or internal-muscle synergies), which serve as a self-organizing operational network^59^. The lower DFA values observed in the present study in right-side dominant participants could only mean the asymmetry in gait control mechanisms due to side-dominance. However, a further increase in their MTC variability (e.g., SD and IQR of MTC; Supplementary Table 8) could mean that right-side dominance appears to be accompanied by an increase in MTC variability as measured by SD and IQR, whereas the scaling DFA properties remain lower. This reciprocal relationship between MTC.

variability and the scale-invariant DFA values of MTC fluctuations could be specific patterns for the neurological process of side-dominance.

### EMG data

**Fig. 4:**
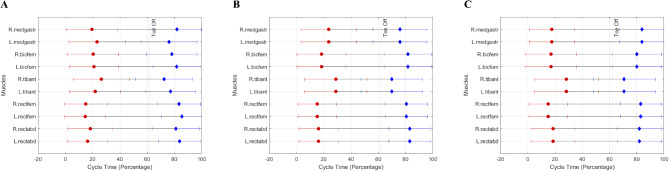
Average Muscle Activation Across All Participants. Minor alterations in the neural control of the muscles on the left (L) and right (R) side during clear glass condition (*Panel A*), left half-field eye patching (*Panel B*), and right half-field eye patching (*Panel C*).

In line with the kinematic data, the results of the EMG measurements showed no major differences in mean EMG activities among the three visual conditions. We had previously proposed that impaired vision while walking could lead to selective up or down-regulated activation of leg and trunk muscles. These changes might serve as a compensation mechanism in response to the decreased visual input. Our results suggest that reducing peripheral vision alone might not be enough to induce changes in lower limb muscle EMG activity patterns or kinematic gait characteristics in healthy young participants. A possible explanation for this could be the long-term neuromuscular adaptations in the active muscles that are involved in walking. Previous reports have shown that the muscle activation pattern remains relatively consistent during a short walking duration, as long as there are no constraints on the walking^75^. Therefore, if reducing vision in either eye does not lead to significant changes, we can expect similar results across the different conditions. It is important to note that using mean EMG activity may hide small differences within the gait cycle that our method was unable to detect. However, based on the visual examination of the mean EMG amplitude data, we do not expect significant differences in EMG amplitude between the conditions using spatial comparative statistics such as statistical parametric mapping. Despite the lack of difference in mean EMG activity of the muscles between visual conditions, we cannot completely exclude the possibility that there were minor alterations in the neural control of the muscles because the reduced visual feedback may require some level of compensation in intra and inter muscle coordination (Fig. 4). We need to take into consideration that the normalization method we used in this study could influence our results and other methods may have yielded different results. Using M-max during walking to normalize EMG signals^76^may more accurately follow the changes in EMG amplitude because a standing value could lead to erroneous conclusions^77^.

Muscles involved in walking are activated at different stages of the step cycle to facilitate intra- and inter-muscular coordination and, hence, a fluent motion. However, it is unknown whether impaired vision would alter the timing of this activation. We did observe small but noticeable differences in the timing of muscle activation and deactivation relative to the clear eye condition, which could indicate minor corrective responses to the eye patch conditions. When walking was performed with the left eye covered on the contralateral side, the right medial gastrocnemius activated later and deactivated earlier, and the right tibialis anterior also activated later. This suggests that the pre-activation period before the heel strike on the clear-eyed side is shorter, potentially resulting in slightly lower joint stiffness and, therefore, less stability. This could be the reason why the right biceps femoris activated earlier, to increase leg stiffness during the braking phase of walking. On the covered eye side, the left tibialis anterior activated earlier and deactivated later, possibly as a compensatory response to the reduced visual feedback on this side in order to maintain ankle joint stability. Biceps femoris on the left leg also activated earlier, possibly due to the absence of full visual feedback on the timing of initial foot contact, leading to an earlier neural command to increase leg stiffness in preparation for ground contact. When walking was performed with the right eye covered, the contralateral lower leg muscles also showed minor differences in muscle activation timings. The left medial gastrocnemius activated later and deactivated earlier, similar to its antagonist, the left tibialis anterior, which also activated later and deactivated earlier. While we did not specifically investigate the effect of visual conditions on muscle timings, the alterations in muscle activation and deactivation timing could be compensatory neural control responses to counteract the lack of visual information during walking.

### Possible underlying mechanisms

In the present study, we applied self-selected speeds to determine the effects of half-field eye patching and side-dominance on kinematic data and muscle activity. Although there have been several other methods, i.e., pre-determined speeds, and statistical control for controlling the walking speeds, however, it was previously suggested that the pre-determined speed control trials have the disadvantage of capturing the unnatural, poor repeatable mechanisms^78^. In addition, considering that a varying speed of self-selected walking in gait experiments could shed light on the biomechanical aspects of walking, we expected to have findings that could not be explained merely by the inter-subject walking speed differences.

One of the possible explanations for the lack of differences between the half-field eye patching conditions is the involvement of healthy participants in the study, i.e., the left or right half-field eye patching is not sufficient enough to induce significant changes in gait characteristics in a healthy population. Another possible explanation for the non-significant effect of half-field eye patching condition on walking gait kinematics and accompanying muscle activation would be that during the familiarization the participants adapted quickly to deploy their natural gait even with reduced visual input. We tried to prevent such adaptation by asking the participants to spend their rest periods with no glasses on and only put them on immediately before starting the familiarization to ensure that it would not confound the results, however, participants in the present study were healthy and young, possibly learning and adapting quickly to such changes that we used to block one of the eyes. Furthermore, it is difficult to draw clear conclusions from our data given that the effects of vision on walking gait control are not well studied. Neither previous studies nor the present one can answer what stage the information gets shared between the two hemispheres. Visual processing is not fully parallel between the two hemispheres; therefore, we hypothesized that blocking one side of vision would only affect the dominant and non-dominant sides differently. However, if the main role of visual input in gait is to determine the distance from the target point in the environment, then gait control should not be affected in healthy participants considering that this function is controlled by both hemispheres.

### Limitations and future perspectives

As always, the study has a few limitations. Although we performed pre-statistical power analyses for sample size calculation, the relatively low sample size could have prevented us from finding significant differences between the visual conditions. Although this cross-over study design seems to be the most appropriate for our aim, it is possible that the healthy participants recruited in this study adapted quickly to deploy their natural gait even with reduced visual input. Overall, the results of the present study are not necessarily generalizable, given that the data are from a small sample of healthy individuals. Second, we determined side-dominance based on handedness and footedness but did not consider participants’ eye-dominance. Moreover, our healthy volunteers walked on a treadmill, which is known to limit the natural gait pattern. Future studies should define side-dominance based on eye-dominance and examine the effects of patch-induced perturbations on gait kinematics and accompanying muscle activation while walking freely in a hallway instead of on a treadmill. Future studies should also clarify if patches obscuring half of the visual field would affect gait kinematics and leg muscle activation of left and right-side-dominant elderly participants or patients with visual or neurological impairments differently.

## Conclusions

The present study aimed to determine if patches obscuring half of the visual field affect left- and right-side dominant individuals’ gait kinematics and leg muscle activation differently. Half-field eye patching condition did not affect participants’ gait kinematics; however, inter-group differences were found on selected gait kinematic parameters. The EMG RMS values were smaller in the non-dominant vs. dominant leg’s (1) medial gastrocnemius during phase II and IV and (2) biceps femoris during phase II. Finally, the endpoint of medial gastrocnemius activation was affected by side-dominance, i.e., its activation ended earlier in the non-dominant leg of right- as compared to left-side dominant participants. Overall, half-field eye patching seems not to affect healthy adults’ kinematic gait parameters and accompanying muscle activation, but the secondary hypotheses of dominant vs. non-dominant limbs and left vs. right-side dominant people seem to have been partially supported.

## Ethics statement

Both verbal and written explanations of the experimental protocol were given in accordance with the declaration of Helsinki. The study was carried out following the recommendations of the University Ethical Committee of the Hungarian University of Sports Science (Approval No. TE-KEB:3:2021). Informed consent was obtained from all subjects and/or their legal guardian(s).

## Electronic supplementary material

Below is the link to the electronic supplementary material.

Supplementary Material 1

## Data Availability

The datasets used and/or analyzed during the current study are presented within the manuscript and/or additional supporting files and are also available from the corresponding author on reasonable request.

## Declarations

### Word count

5689 words.
