# Supplementary material for: Side dominance and eye patches obscuring half of the visual field do not affect walking kinematics
Source: Sci Rep. 2025 Feb 20;15:6189. doi: 10.1038/s41598-025-90936-x (PMC11842710; doi:10.1038/s41598-025-90936-x)
Supplement: Supplementary file 1 — Supplementary Material 1 [file 41598_2025_90936_MOESM1_ESM.docx]

*Supplementary material*

**Title:** Walking kinematics is not affected by patches obscuring half of the visual field, regardless of side dominance

**Journal name:** nature Scientific Reports

**Authors:** János Négyesi^1, 2, 3 *, †^, Bálint Kovács^4, 1, †^, Bálint Petró^5^, Diane Nabil Salman^6^, Ahsan Khandoker^6^, Péter Katona^1^, Mostafa Mohamed Moussa^6^, Tibor Hortobágyi^1, 7, 8, 9^, Kristóf Rácz^5^, Zsófia Pálya^5^, László Grand^10^, Rita Mária Kiss^5^, Ryoichi Nagatomi^11^

**Affiliations:** *^1^Department of Kinesiology, Hungarian University of Sports Science, Budapest, Hungary; ^2^Neurocognitive Research Center, Nyírő Gyula National Institute of Psychiatry, and Addictology, Budapest, Hungary; ^3^CRU Hungary Kft., Budapest, Hungary; ^4^Ningbo University, Faculty of Sport Science, Ningbo, China; ^5^Faculty of Mechanical Engineering, Department of Mechatronics, Optics and Mechanical Engineering Informatics, Budapest University of Technology and Economics, Budapest, Hungary; ^6^Biomedical Engineering Department, Khalifa University, Abu Dhabi, UAE; ^7^Department of Neurology, Somogy County Kaposi Mór Teaching Hospital, 7400, Kaposvár, Hungary; ^8^Department of Sport Biology, Institute of Sport Sciences and Physical Education, University of Pécs, Pécs, Hungary; ^9^Center for Human Movement Sciences, University of Groningen, University Medical Center Groningen, Groningen, The Netherlands;; ^10^Faculty of Information Technology, Pázmány Péter Catholic University, Budapest, Hungary; ^11^Designing Future Health Initiative (DFHI), Promotion Office of Strategic Innovation, Tohoku University, Sendai, Japan*

**Corresponding author:**

János Négyesi

e-mail: [negyesi.janos@tf.hu](mailto:negyesi.janos@tf.hu)

| **Supplementary Table 1:** Summary of gait parameters and motion capture analysis variables | | | | | | | | |
| --- | --- | --- | --- | --- | --- | --- | --- | --- |
| **Parameter** |  | **Abbreviation** |  | **Units** |  | **Definition** |  | **Variables** |
|  |  |  |  |  |  |  |  |  |
| *Spatiotemporal gait parameters* | | | | | | | | |
| Stride time |  | stride_time |  | s |  | Time between two consecutive heel-strikes of the same foot. |  | - Mean - Median - Mode - Min - Max - SD - RMS - Q1 - Q3 - IQR - Skewness - Kurtosis   DFA* (overall alpha, alpha 1 and alpha 2) |
| Stride length |  | stride_length |  | cm |  | Distance between two consecutive heel-strike locations of the same foot along the direction of progression. |  |  |
| Stride speed |  | stride_speed |  | m/s |  | stride_length / stride_time |  |  |
| Step time |  | step_time |  | s |  | Time between the heel-strike of one foot and the following heel-strike of the opposite foot. |  |  |
| Step length |  | step_length |  | cm |  | Distance between the heel-strike location of one foot and the location of the following heel-strike of the opposite foot, along the direction of progression. |  |  |
| Step width |  | step_width |  | cm |  | Distance between the heel-strike location of one foot and the location of the following heel-strike of the opposite foot, in the medio-lateral direction. |  |  |
| Minimum toe clearance |  | MTC |  | cm |  | Minimum vertical distance between toes and ground during the swing phase of a gait cycle. |  |  |
| Horizontal velocity at MTC |  | velMTC |  | m/s |  | Horizontal velocity of foot at time of MTC. |  |  |
| Cadence |  | cadence |  | steps/min |  | Step frequency expressed as number of steps per minute. |  | N/A |
|  |  |  |  |  |  |  |  |  |
| *Joint angle kinematic gait parameters* | | | | | | | | |
| Hip flexion |  | hip_flexion |  | degrees |  | Hip angle reported by the lower body OpenSim model based on Delp et al. (2007) [1]. |  | Range of motion (ROM), SD, peak flexion and peak extension |
| Knee angle |  | knee_angle |  | degrees |  | Knee angle reported by the lower body OpenSim model based on Delp et al. (2007) [1]. |  | ROM, SD, initial contact flexion, peak flexion, midstance minimum flexion |
| Ankle angle |  | ankle_angle |  | degrees |  | Ankle angle reported by the lower body OpenSim model based on Delp et al. (2007) [1]. |  | ROM, SD, peak flexion, peak extension, initial contact flexion |
|  |  |  |  |  |  |  |  |  |
| *Lateral pelvic translation parameters* | | | | | | | | |
| Average lateral pelvic translation |  | avg_pelvis_tz |  | mm |  | Change in average pelvis medial-lateral translation between gait cycles. |  | Trend (linear regression slope and p-value), mean, median, mode, SD, Skewness and Kurtosis |
| IQR of lateral pelvic translation |  | iqr_pelvis_tz |  | N/A |  | Change in IQR of pelvis medial-lateral translation between gait cycles. |  | Number of corrective cycles = number of times the IQR is below the 2^nd^ percentile or above the 98th percentile |
| Pelvic butterfly plot |  | butterfly_vars |  | N/A |  | Plot showing average medial-lateral pelvis displacement during one gait cycle by plotting average anterior-posterior pelvic movement against lateral pelvic movement with correction for displacement in the line of progression. Representation of pelvic lateral movement as viewed from top. |  | Pelvic lateral displacement (PLD): absolute peak to peak displacement of the pelvis in the medial lateral direction during a gait cycle (stride)  Area: area under the curve of the butterfly plot for each side (left and right) |
| *Window size used for the DFA is:   - For overall alpha: 4 to N/4 - For alpha1: N/52 to N/20 - For alpha2: N/8 to N/4   Where N is total number of strides in the recording | | | | | | | | |

| **Supplementary Table 2:** Kinematic data of stride time | | | | | | | | | |
| --- | --- | --- | --- | --- | --- | --- | --- | --- | --- |
|  |  | **Clear glass** | |  | **Left half-field eye patching** | |  | **Right half-field eye patching** | |
|  |  | *D* | *ND* |  | *D* | *ND* |  | *D* | *ND* |
| Mean (s) |  |  |  |  |  |  |  |  |  |
| *Right-side dominant* |  | 1.17  (0.11) | 1.17  (0.11) |  | 1.16  (0.11) | 1.16  (0.11) |  | 1.16  (0.10) | 1.16  (0.10) |
| *Left-side dominant* |  | 1.18  (0.14) | 1.18  (0.14) |  | 1.19  (0.14) | 1.19  (0.14) |  | 1.20  (0.13) | 1.20  (0.13) |
|  |  |  |  |  |  |  |  |  |  |
| Min (s) |  |  |  |  |  |  |  |  |  |
| *Right-side dominant* |  | 1.12  (0.10) | 1.12  (0.10) |  | 1.11  (0.10) | 1.11  (0.10) |  | 1.11  (0.09) | 1.11  (0.09) |
| *Left-side dominant* |  | 1.14  (0.12) | 1.14  (0.12) |  | 1.15  (0.13) | 1.14  (0.13) |  | 1.15  (0.12) | 1.14  (0.12) |
|  |  |  |  |  |  |  |  |  |  |
| Max (s) |  |  |  |  |  |  |  |  |  |
| *Right-side dominant* |  | 1.22  (0.13) | 1.22  (0.13) |  | 1.21  (0.13) | 1.21  (0.13) |  | 1.21  (0.12) | 1.21  (0.12) |
| *Left-side dominant* |  | 1.23  (0.14) | 1.23  (0.15) |  | 1.24  (0.15) | 1.24  (0.16) |  | 1.24  (0.14) | 1.24  (0.14) |
|  |  |  |  |  |  |  |  |  |  |
| SD (s) |  |  |  |  |  |  |  |  |  |
| *Right-side dominant* |  | 0.02  (0.01) | 0.02  (0.01) |  | 0.02  (0.01) | 0.02  (0.01) |  | 0.02  (0.01) | 0.02  (0.01) |
| *Left-side dominant* |  | 0.02  (0.00) | 0.02  (0.00) |  | 0.02  (0.01) | 0.02  (0.01) |  | 0.02  (0.01) | 0.02  (0.01) |
|  |  |  |  |  |  |  |  |  |  |
| RMS (s) |  |  |  |  |  |  |  |  |  |
| *Right-side dominant* |  | 1.17  (0.11) | 1.17  (0.11) |  | 1.16  (0.11) | 1.16  (0.11) |  | 1.16  (0.10) | 1.16  (0.10) |
| *Left-side dominant* |  | 1.19  (0.14) | 1.19  (0.14) |  | 1.19  (0.14) | 1.19  (0.14) |  | 1.20  (0.13) | 1.20  (0.13) |
|  |  |  |  |  |  |  |  |  |  |
| Median (s) |  |  |  |  |  |  |  |  |  |
| *Right-side dominant* |  | 1.17  (0.11) | 1.17  (0.11) |  | 1.16  (0.11) | 1.16  (0.11) |  | 1.16  (0.10) | 1.16  (0.10) |
| *Left-side dominant* |  | 1.19  (0.13) | 1.19  (0.13) |  | 1.19  (0.14) | 1.19  (0.14) |  | 1.20  (0.13) | 1.20  (0.13) |
|  |  |  |  |  |  |  |  |  |  |
| Q1 (s) |  |  |  |  |  |  |  |  |  |
| *Right-side dominant* |  | 1.16  (0.11) | 1.16  (0.11) |  | 1.15  (0.11) | 1.15  (0.11) |  | 1.15  (0.10) | 1.15  (0.10) |
| *Left-side dominant* |  | 1.18  (0.13) | 1.18  (0.13) |  | 1.18  (0.14) | 1.18  (0.14) |  | 1.18  (0.13) | 1.18  (0.13) |
|  |  |  |  |  |  |  |  |  |  |
| Q3 (s) |  |  |  |  |  |  |  |  |  |
| *Right-side dominant* |  | 1.18  (0.11) | 1.18  (0.11) |  | 1.17  (0.11) | 1.17  (0.11) |  | 1.17  (0.11) | 1.17  (0.11) |
| *Left-side dominant* |  | 1.20  (0.14) | 1.20  (0.14) |  | 1.20  (0.14) | 1.20  (0.14) |  | 1.21  (0.13) | 1.21  (0.13) |
|  |  |  |  |  |  |  |  |  |  |
| IQR (s) |  |  |  |  |  |  |  |  |  |
| *Right-side dominant* |  | 0.02  (0.01) | 0.02  (0.01) |  | 0.02  (0.01) | 0.02  (0.01) |  | 0.02  (0.01) | 0.02  (0.01) |
| *Left-side dominant* |  | 0.02  (0.01) | 0.02  (0.01) |  | 0.02  (0.01) | 0.02  (0.01) |  | 0.02  (0.01) | 0.02  (0.01) |
|  |  |  |  |  |  |  |  |  |  |
| Mode (s) |  |  |  |  |  |  |  |  |  |
| *Right-side dominant* |  | 1.17  (0.11) | 1.17  (0.11) |  | 1.16  (0.11) | 1.16  (0.11) |  | 1.16  (0.11) | 1.16  (0.10) |
| *Left-side dominant* |  | 1.19  (0.14) | 1.18  (0.13) |  | 1.19  (0.14) | 1.19  (0.14) |  | 1.20  (0.14) | 1.20  (0.13) |
|  |  |  |  |  |  |  |  |  |  |
| Skewness (s) |  |  |  |  |  |  |  |  |  |
| *Right-side dominant* |  | 0.05  (0.30) | 0.04  (0.27) |  | -0.07  (0.37) | -0.07  (0.37) |  | -0.01  (0.28) | -0.07  (0.26) |
| *Left-side dominant* |  | -0.09  (0.17) | -0.07  (0.20) |  | 0.06  (0.27) | -0.01  (0.26) |  | -0.11  (0.21) | -0.14  (0.21) |
|  |  |  |  |  |  |  |  |  |  |
| Kurtosis (s) * |  |  |  |  |  |  |  |  |  |
| *Right-side dominant* |  | 3.07  (0.43) | 3.06  (0.18) |  | 3.36  (0.71) | 3.24  (0.33) |  | 3.30  (0.36) | 3.27  (0.39) |
| *Left-side dominant* |  | 3.06  (0.30) | 3.04  (0.21) |  | 3.00  (0.20) | 3.02  (0.23) |  | 2.93  (0.44) | 2.92  (0.31) |
|  |  |  |  |  |  |  |  |  |  |
| DFA - overall (s) |  |  |  |  |  |  |  |  |  |
| *Right-side dominant* |  | 0.76  (0.17) | 0.76  (0.17) |  | 0.77  (0.17) | 0.76  (0.17) |  | 0.78  (0.15) | 0.79  (0.16) |
| *Left-side dominant* |  | 0.85  (0.13) | 0.85  (0.12) |  | 0.78  (0.09) | 0.77  (0.09) |  | 0.80  (0.12) | 0.79  (0.11) |
|  |  |  |  |  |  |  |  |  |  |
| DFA – alpha1 (s) |  |  |  |  |  |  |  |  |  |
| *Right-side dominant* |  | 0.73  (0.20) | 0.69  (0.16) |  | 0.69  (0.17) | 0.69  (0.19) |  | 0.71  (0.19) | 0.70  (0.22) |
| *Left-side dominant* |  | 0.74  (0.11) | 0.73  (0.10) |  | 0.65  (0.13) | 0.65  (0.12) |  | 0.70  (0.08) | 0.67  (0.07) |
|  |  |  |  |  |  |  |  |  |  |
| DFA – alpha2 (s) * |  |  |  |  |  |  |  |  |  |
| *Right-side dominant* |  | 0.80  (0.46) | 0.79  (0.46) |  | 0.89  (0.34) | 0.86  (0.33) |  | 0.78  (0.24) | 0.78  (0.23) |
| *Left-side dominant* |  | 1.07  (0.33) | 1.07  (0.33) |  | 0.95  (0.36) | 0.95  (0.37) |  | 0.93  (0.29) | 0.91  (0.28) |
| Values are mean (SD) of each variable.  D: dominant leg, DFA: detrended fluctuation analysis; IQR: interquartile range; ND: non-dominant leg; Q1: first quarter; Q3: third quarter; RMS: root mean square; SD: standard deviation  * indicates differences between the left and right-side dominant participants | | | | | | | | | |

| **Supplementary Table 3:** Kinematic data of stride length | | | | | | | | | |
| --- | --- | --- | --- | --- | --- | --- | --- | --- | --- |
|  |  | **Clear glass** | |  | **Left half-field eye patching** | |  | **Right half-field eye patching** | |
|  |  | *D* | *ND* |  | *D* | *ND* |  | *D* | *ND* |
| Mean (cm) |  |  |  |  |  |  |  |  |  |
| *Right-side dominant* |  | 112.7  (16.6) | 112.7  (16.6) |  | 112.0  (16.1) | 112.0  (16.1) |  | 112.0  (16.9) | 112.0  (16.9) |
| *Left-side dominant* |  | 113.2  (12.0) | 113.2  (12.0) |  | 113.6  (11.8) | 113.6  (11.8) |  | 114.2  (11.9) | 114.2  (11.9) |
|  |  |  |  |  |  |  |  |  |  |
| Min (cm) |  |  |  |  |  |  |  |  |  |
| *Right-side dominant* |  | 106.6  (16.0) | 106.2  (15.5) |  | 105.7  (14.8) | 106.0  (15.6) |  | 106.5  (17.4) | 106.5  (17.4) |
| *Left-side dominant* |  | 107.5  (12.2) | 107.7  (12.3) |  | 108.3  (11.1) | 108.3  (11.0) |  | 107.5  (13.2) | 108.2  (12.8) |
|  |  |  |  |  |  |  |  |  |  |
| Max (cm) |  |  |  |  |  |  |  |  |  |
| *Right-side dominant* |  | 117.6  (16.4) | 118.0  (16.4) |  | 117.2  (16.2) | 117.7  (16.0) |  | 117.6  (16.5) | 117.2  (16.6) |
| *Left-side dominant* |  | 118.6  (11.7) | 118.9  (11.9) |  | 118.6  (11.7) | 119.2  (11.8) |  | 119.8  (12.0) | 119.3  (12.0) |
|  |  |  |  |  |  |  |  |  |  |
| SD (cm) |  |  |  |  |  |  |  |  |  |
| *Right-side dominant* |  | 1.82  (0.48) | 1.81  (0.46) |  | 1.83  (0.47) | 1.84  (0.50) |  | 1.81  (0.49) | 1.81  (0.48) |
| *Left-side dominant* |  | 1.76  (0.40) | 1.81  (0.42) |  | 1.78  (0.37) | 1.82  (0.41) |  | 1.93  (0.57) | 1.93  (0.59) |
|  |  |  |  |  |  |  |  |  |  |
| RMS (cm) |  |  |  |  |  |  |  |  |  |
| *Right-side dominant* |  | 112.7  (16.6) | 112.7  (16.6) |  | 112.0  (16.1) | 112.0  (16.1) |  | 112.1  (16.9) | 112.1  (16.9) |
| *Left-side dominant* |  | 113.2  (12.0) | 113.2  (12.0) |  | 113.6  (11.8) | 113.6  (11.8) |  | 114.2  (11.9) | 114.2  (11.9) |
|  |  |  |  |  |  |  |  |  |  |
| Median (cm) |  |  |  |  |  |  |  |  |  |
| *Right-side dominant* |  | 112.8  (16.6) | 112.7  (16.6) |  | 112.1  (16.2) | 112.1  (16.2) |  | 112.0  (17.0) | 112.0  (16.9) |
| *Left-side dominant* |  | 113.3  (12.0) | 113.2  (12.0) |  | 113.6  (11.8) | 113.6  (11.9) |  | 114.2  (11.9) | 114.3  (11.9) |
|  |  |  |  |  |  |  |  |  |  |
| Q1 (cm) |  |  |  |  |  |  |  |  |  |
| *Right-side dominant* |  | 111.5  (16.6) | 111.5  (16.6) |  | 110.9  (16.2) | 110.8  (16.2) |  | 110.9  (17.0) | 110.9  (17.0) |
| *Left-side dominant* |  | 112.1  (12.0) | 112.1  (12.0) |  | 112.5  (11.8) | 112.5  (11.8) |  | 113.0  (12.0) | 112.9  (12.0) |
|  |  |  |  |  |  |  |  |  |  |
| Q3 (cm) |  |  |  |  |  |  |  |  |  |
| *Right-side dominant* |  | 113.9  (16.6) | 113.9  (16.6) |  | 113.2  (16.1) | 113.2  (16.1) |  | 113.2  (16.9) | 113.3  (16.9) |
| *Left-side dominant* |  | 114.4  (12.0) | 114.5  (12.0) |  | 114.8  (11.8) | 114.9  (11.9) |  | 115.5  (11.8) | 115.5  (11.8) |
|  |  |  |  |  |  |  |  |  |  |
| IQR (cm) |  |  |  |  |  |  |  |  |  |
| *Right-side dominant* |  | 2.37  (0.70) | 2.38  (0.69) |  | 2.32  (0.49) | 2.42  (0.53) |  | 2.35  (0.67) | 2.38  (0.76) |
| *Left-side dominant* |  | 2.27  (0.48) | 2.38  (0.51) |  | 2.37  (0.59) | 2.40  (0.57) |  | 2.53  (0.93) | 2.62  (1.03) |
|  |  |  |  |  |  |  |  |  |  |
| Mode (cm) |  |  |  |  |  |  |  |  |  |
| *Right-side dominant* |  | 106.6  (16.0) | 106.2  (15.5) |  | 105.7  (14.8) | 106.0  (15.6) |  | 106.5  (17.4) | 106.5  (17.4) |
| *Left-side dominant* |  | 107.5  (12.2) | 107.7  (12.3) |  | 108.3  (11.1) | 108.3  (11.0) |  | 107.5  (13.2) | 108.2  (12.8) |
|  |  |  |  |  |  |  |  |  |  |
| Skewness (cm) |  |  |  |  |  |  |  |  |  |
| *Right-side dominant* |  | -0.20  (0.39) | -0.23  (0.54) |  | -0.24  (0.71) | -0.17  (0.64) |  | -0.04  (0.26) | -0.12  (0.27) |
| *Left-side dominant* |  | -0.07  (0.11) | -0.04  (0.30) |  | -0.14  (0.19) | -0.05  (0.33) |  | -0.24  (0.41) | -0.18  (0.25) |
|  |  |  |  |  |  |  |  |  |  |
| Kurtosis (cm) |  |  |  |  |  |  |  |  |  |
| *Right-side dominant* |  | 3.67  (1.65) | 4.21  (3.18) |  | 4.22  (3.97) | 4.04  (2.80) |  | 3.40  (0.45) | 3.32  (0.60) |
| *Left-side dominant* |  | 3.39  (0.63) | 3.54  (0.95) |  | 3.22  (0.29) | 3.31  (0.79) |  | 3.95  (2.16) | 3.34  (0.63) |
|  |  |  |  |  |  |  |  |  |  |
| DFA - overall (cm) |  |  |  |  |  |  |  |  |  |
| *Right-side dominant* |  | 0.70  (0.13) | 0.71  (0.14) |  | 0.71  (0.11) | 0.71  (0.11) |  | 0.70  (0.13) | 0.70  (0.14) |
| *Left-side dominant* |  | 0.78  (0.14) | 0.77  (0.14) |  | 0.74  (0.10) | 0.73  (0.09) |  | 0.73  (0.12) | 0.73  (0.13) |
|  |  |  |  |  |  |  |  |  |  |
| DFA – alpha1 (cm) |  |  |  |  |  |  |  |  |  |
| *Right-side dominant* |  | 0.69  (0.12) | 0.69  (0.13) |  | 0.63  (0.08) | 0.61  (0.07) |  | 0.67  (0.11) | 0.68  (0.12) |
| *Left-side dominant* |  | 0.70  (0.10) | 0.69  (0.11) |  | 0.65  (0.08) | 0.65  (0.06) |  | 0.61  (0.10) | 0.63  (0.08) |
|  |  |  |  |  |  |  |  |  |  |
| DFA – alpha2 (cm) |  |  |  |  |  |  |  |  |  |
| *Right-side dominant* |  | 0.73  (0.45) | 0.74  (0.47) |  | 0.82  (0.31) | 0.82  (0.31) |  | 0.75  (0.25) | 0.75  (0.25) |
| *Left-side dominant* |  | 0.94  (0.34) | 0.95  (0.35) |  | 0.90  (0.37) | 0.92  (0.39) |  | 0.99  (0.40) | 0.99  (0.41) |
| Values are mean (SD) of each variable.  D: dominant leg, DFA: detrended fluctuation analysis; IQR: interquartile range; ND: non-dominant leg; Q1: first quarter; Q3: third quarter; RMS: root mean square; SD: standard deviation | | | | | | | | | |

| **Supplementary Table 4:** Kinematic data of stride speed | | | | | | | | | |
| --- | --- | --- | --- | --- | --- | --- | --- | --- | --- |
|  |  | **Clear glass** | |  | **Left half-field eye patching** | |  | **Right half-field eye patching** | |
|  |  | *D* | *ND* |  | *D* | *ND* |  | *D* | *ND* |
| Mean (m/s) |  |  |  |  |  |  |  |  |  |
| *Right-side dominant* |  | 0.98  (0.22) | 0.98  (0.22) |  | 0.98  (0.21) | 0.98  (0.21) |  | 0.98  (0.22) | 0.98  (0.22) |
| *Left-side dominant* |  | 0.97  (0.15) | 0.97  (0.15) |  | 0.97  (0.15) | 0.97  (0.15) |  | 0.97  (0.15) | 0.97  (0.15) |
|  |  |  |  |  |  |  |  |  |  |
| Min (m/s) |  |  |  |  |  |  |  |  |  |
| *Right-side dominant* |  | 0.93  (0.21) | 0.93  (0.21) |  | 0.93  (0.20) | 0.94  (0.21) |  | 0.94  (0.22) | 0.94  (0.22) |
| *Left-side dominant* |  | 0.92  (0.15) | 0.92  (0.14) |  | 0.92  (0.15) | 0.92  (0.15) |  | 0.91  (0.15) | 0.92  (0.15) |
|  |  |  |  |  |  |  |  |  |  |
| Max (m/s) |  |  |  |  |  |  |  |  |  |
| *Right-side dominant* |  | 1.03  (0.22) | 1.02  (0.22) |  | 1.03  (0.23) | 1.03  (0.22) |  | 1.03  (0.21) | 1.03  (0.21) |
| *Left-side dominant* |  | 1.01  (0.15) | 1.02  (0.15) |  | 1.01  (0.16) | 1.02  (0.15) |  | 1.01  (0.15) | 1.02  (0.15) |
|  |  |  |  |  |  |  |  |  |  |
| SD (m/s) |  |  |  |  |  |  |  |  |  |
| *Right-side dominant* |  | 0.02  (0.00) | 0.01  (0.00) |  | 0.02  (0.00) | 0.01  (0.00) |  | 0.01  (0.00) | 0.01  (0.00) |
| *Left-side dominant* |  | 0.01  (0.00) | 0.02  (0.00) |  | 0.01  (0.00) | 0.02  (0.00) |  | 0.01  (0.00) | 0.02  (0.00) |
|  |  |  |  |  |  |  |  |  |  |
| RMS (m/s) |  |  |  |  |  |  |  |  |  |
| *Right-side dominant* |  | 0.98  (0.22) | 0.98  (0.22) |  | 0.98  (0.21) | 0.98  (0.21) |  | 0.98  (0.22) | 0.98  (0.22) |
| *Left-side dominant* |  | 0.97  (0.15) | 0.97  (0.15) |  | 0.97  (0.15) | 0.97  (0.15) |  | 0.97  (0.15) | 0.97  (0.15) |
|  |  |  |  |  |  |  |  |  |  |
| Median (m/s) |  |  |  |  |  |  |  |  |  |
| *Right-side dominant* |  | 0.98  (0.22) | 0.98  (0.22) |  | 0.98  (0.21) | 0.98  (0.22) |  | 0.98  (0.22) | 0.98  (0.22) |
| *Left-side dominant* |  | 0.97  (0.15) | 0.97  (0.15) |  | 0.97  (0.15) | 0.97  (0.15) |  | 0.97  (0.15) | 0.97  (0.15) |
|  |  |  |  |  |  |  |  |  |  |
| Q1 (m/s) |  |  |  |  |  |  |  |  |  |
| *Right-side dominant* |  | 0.97  (0.22) | 0.97  (0.22) |  | 0.97  (0.21) | 0.97  (0.21) |  | 0.97  (0.22) | 0.97  (0.22) |
| *Left-side dominant* |  | 0.96  (0.15) | 0.96  (0.15) |  | 0.96  (0.15) | 0.96  (0.15) |  | 0.96  (0.15) | 0.96  (0.15) |
|  |  |  |  |  |  |  |  |  |  |
| Q3 (m/s) |  |  |  |  |  |  |  |  |  |
| *Right-side dominant* |  | 0.99  (0.22) | 0.99  (0.22) |  | 0.99  (0.22) | 0.99  (0.22) |  | 0.99  (0.22) | 0.99  (0.22) |
| *Left-side dominant* |  | 0.98  (0.15) | 0.98  (0.15) |  | 0.98  (0.15) | 0.98  (0.15) |  | 0.98  (0.15) | 0.98  (0.15) |
|  |  |  |  |  |  |  |  |  |  |
| IQR (m/s) |  |  |  |  |  |  |  |  |  |
| *Right-side dominant* |  | 0.02  (0.00) | 0.02  (0.00) |  | 0.02  (0.00) | 0.02  (0.00) |  | 0.02  (0.00) | 0.02  (0.00) |
| *Left-side dominant* |  | 0.02  (0.00) | 0.02  (0.00) |  | 0.02  (0.00) | 0.02  (0.00) |  | 0.02  (0.00) | 0.02  (0.00) |
|  |  |  |  |  |  |  |  |  |  |
| Mode (m/s) |  |  |  |  |  |  |  |  |  |
| *Right-side dominant* |  | 0.93  (0.21) | 0.93  (0.21) |  | 0.93  (0.20) | 0.94  (0.21) |  | 0.94  (0.22) | 0.94  (0.22) |
| *Left-side dominant* |  | 0.92  (0.15) | 0.92  (0.14) |  | 0.92  (0.15) | 0.92  (0.15) |  | 0.91  (0.15) | 0.92  (0.15) |
|  |  |  |  |  |  |  |  |  |  |
| Skewness (m/s) |  |  |  |  |  |  |  |  |  |
| *Right-side dominant* |  | -0.04  (0.32) | -0.05  (0.34) |  | 0.05  (0.46) | 0.02  (0.53) |  | 0.12  (0.34) | 0.03  (0.36) |
| *Left-side dominant* |  | 0.13  (0.24) | -0.01  (0.66) |  | 0.00  (0.39) | 0.16  (0.48) |  | -0.17  (0.67) | 0.00  (0.30) |
|  |  |  |  |  |  |  |  |  |  |
| Kurtosis (m/s) |  |  |  |  |  |  |  |  |  |
| *Right-side dominant* |  | 3.56  (1.02) | 3.90  (2.11) |  | 4.01  (2.66) | 4.22  (3.10) |  | 3.81  (1.34) | 3.75  (1.13) |
| *Left-side dominant* |  | 3.44  (0.73) | 4.37  (2.71) |  | 3.95  (1.88) | 4.06  (2.81) |  | 4.78  (2.61) | 3.56  (0.87) |
|  |  |  |  |  |  |  |  |  |  |
| DFA - overall (m/s) * |  |  |  |  |  |  |  |  |  |
| *Right-side dominant* |  | 0.37  (0.06) | 0.39  (0.08) |  | 0.35  (0.07) | 0.37  (0.07) |  | 0.34  (0.06) | 0.35  (0.05) |
| *Left-side dominant* |  | 0.43  (0.06) | 0.42  (0.08) |  | 0.40  (0.05) | 0.39  (0.05) |  | 0.40  (0.09) | 0.38  (0.07) |
|  |  |  |  |  |  |  |  |  |  |
| DFA – alpha1 (m/s) |  |  |  |  |  |  |  |  |  |
| *Right-side dominant* |  | 0.47  (0.11) | 0.46  (0.09) |  | 0.42  (0.09) | 0.45  (0.07) |  | 0.45  (0.08) | 0.46  (0.08) |
| *Left-side dominant* |  | 0.48  (0.11) | 0.46  (0.13) |  | 0.49  (0.12) | 0.51  (0.10) |  | 0.46  (0.13) | 0.48  (0.14) |
|  |  |  |  |  |  |  |  |  |  |
| DFA – alpha2 (m/s) |  |  |  |  |  |  |  |  |  |
| *Right-side dominant* |  | 0.34  (0.11) | 0.37  (0.12) |  | 0.36  (0.15) | 0.37  (0.14) |  | 0.31  (0.12) | 0.32  (0.12) |
| *Left-side dominant* |  | 0.31  (0.13) | 0.32  (0.14) |  | 0.36  (0.18) | 0.34  (0.18) |  | 0.33  (0.14) | 0.30  (0.13) |
| Values are mean (SD) of each variable.  D: dominant leg, DFA: detrended fluctuation analysis; IQR: interquartile range; ND: non-dominant leg; Q1: first quarter; Q3: third quarter; RMS: root mean square; SD: standard deviation  * indicates differences between the left and right-side dominant participants | | | | | | | | | |

| **Supplementary Table 5:** Kinematic data of step time | | | | | | | | | |
| --- | --- | --- | --- | --- | --- | --- | --- | --- | --- |
|  |  | **Clear glass** | |  | **Left half-field eye patching** | |  | **Right half-field eye patching** | |
|  |  | *D* | *ND* |  | *D* | *ND* |  | *D* | *ND* |
| Mean (s) |  |  |  |  |  |  |  |  |  |
| *Right-side dominant* |  | 0.58  (0.06) | 0.58  (0.05) |  | 0.58  (0.06) | 0.58  (0.05) |  | 0.58  (0.05) | 0.58  (0.05) |
| *Left-side dominant* |  | 0.59  (0.07) | 0.59  (0.07) |  | 0.60  (0.07) | 0.59  (0.07) |  | 0.60  (0.07) | 0.60  (0.06) |
|  |  |  |  |  |  |  |  |  |  |
| Min (s) |  |  |  |  |  |  |  |  |  |
| *Right-side dominant* |  | 0.55  (0.05) | 0.55  (0.05) |  | 0.55  (0.05) | 0.55  (0.05) |  | 0.54  (0.05) | 0.55  (0.04) |
| *Left-side dominant* |  | 0.56  (0.07) | 0.56  (0.06) |  | 0.57  (0.07) | 0.56  (0.07) |  | 0.57  (0.06) | 0.56  (0.06) |
|  |  |  |  |  |  |  |  |  |  |
| Max (s) |  |  |  |  |  |  |  |  |  |
| *Right-side dominant* |  | 0.62  (0.07) | 0.62  (0.06) |  | 0.62  (0.07) | 0.61  (0.06) |  | 0.61  (0.06) | 0.61  (0.06) |
| *Left-side dominant* |  | 0.63  (0.08) | 0.62  (0.07) |  | 0.63  (0.08) | 0.62  (0.08) |  | 0.63  (0.07) | 0.63  (0.07) |
|  |  |  |  |  |  |  |  |  |  |
| SD (s) |  |  |  |  |  |  |  |  |  |
| *Right-side dominant* |  | 0.01  (0.00) | 0.01  (0.00) |  | 0.01  (0.00) | 0.01  (0.00) |  | 0.01  (0.00) | 0.01  (0.00) |
| *Left-side dominant* |  | 0.01  (0.00) | 0.01  (0.00) |  | 0.01  (0.00) | 0.01  (0.00) |  | 0.01  (0.00) | 0.01  (0.00) |
|  |  |  |  |  |  |  |  |  |  |
| RMS (s) |  |  |  |  |  |  |  |  |  |
| *Right-side dominant* |  | 0.58  (0.06) | 0.58  (0.05) |  | 0.58  (0.06) | 0.58  (0.05) |  | 0.58  (0.05) | 0.58  (0.05) |
| *Left-side dominant* |  | 0.59  (0.07) | 0.59  (0.07) |  | 0.60  (0.07) | 0.59  (0.07) |  | 0.60  (0.07) | 0.60  (0.06) |
|  |  |  |  |  |  |  |  |  |  |
| Median (s) |  |  |  |  |  |  |  |  |  |
| *Right-side dominant* |  | 0.58  (0.06) | 0.58  (0.05) |  | 0.58  (0.06) | 0.58  (0.05) |  | 0.58  (0.05) | 0.58  (0.05) |
| *Left-side dominant* |  | 0.60  (0.07) | 0.59  (0.07) |  | 0.60  (0.07) | 0.59  (0.07) |  | 0.60  (0.07) | 0.60  (0.06) |
|  |  |  |  |  |  |  |  |  |  |
| Q1 (s) |  |  |  |  |  |  |  |  |  |
| *Right-side dominant* |  | 0.58  (0.06) | 0.58  (0.05) |  | 0.57  (0.06) | 0.57  (0.05) |  | 0.57  (0.05) | 0.57  (0.05) |
| *Left-side dominant* |  | 0.59  (0.07) | 0.58  (0.07) |  | 0.59  (0.07) | 0.59  (0.07) |  | 0.59  (0.06) | 0.59  (0.06) |
|  |  |  |  |  |  |  |  |  |  |
| Q3 (s) |  |  |  |  |  |  |  |  |  |
| *Right-side dominant* |  | 0.59  (0.06) | 0.59  (0.06) |  | 0.59  (0.06) | 0.59  (0.05) |  | 0.59  (0.06) | 0.59  (0.05) |
| *Left-side dominant* |  | 0.60  (0.07) | 0.60  (0.07) |  | 0.60  (0.07) | 0.60  (0.07) |  | 0.61  (0.07) | 0.60  (0.07) |
|  |  |  |  |  |  |  |  |  |  |
| IQR (s) |  |  |  |  |  |  |  |  |  |
| *Right-side dominant* |  | 0.02  (0.01) | 0.01  (0.00) |  | 0.01  (0.01) | 0.01  (0.01) |  | 0.01  (0.01) | 0.02  (0.01) |
| *Left-side dominant* |  | 0.01  (0.00) | 0.01  (0.01) |  | 0.01  (0.01) | 0.01  (0.01) |  | 0.01  (0.01) | 0.02  (0.01) |
|  |  |  |  |  |  |  |  |  |  |
| Mode (s) |  |  |  |  |  |  |  |  |  |
| *Right-side dominant* |  | 0.58  (0.06) | 0.58  (0.05) |  | 0.58  (0.06) | 0.58  (0.05) |  | 0.58  (0.05) | 0.58  (0.05) |
| *Left-side dominant* |  | 0.60  (0.07) | 0.59  (0.07) |  | 0.60  (0.07) | 0.59  (0.07) |  | 0.60  (0.07) | 0.60  (0.06) |
|  |  |  |  |  |  |  |  |  |  |
| Skewness (s) |  |  |  |  |  |  |  |  |  |
| *Right-side dominant* |  | 0.04  (0.15) | 0.02  (0.22) |  | -0.05  (0.36) | -0.06  (0.30) |  | 0.02  (0.20) | 0.01  (0.25) |
| *Left-side dominant* |  | -0.02  (0.17) | -0.06  (0.24) |  | 0.10  (0.28) | 0.00  (0.23) |  | -0.12  (0.23) | -0.04  (0.24) |
|  |  |  |  |  |  |  |  |  |  |
| Kurtosis (s) * |  |  |  |  |  |  |  |  |  |
| *Right-side dominant* |  | 3.00  (0.23) | 3.15  (0.31) |  | 3.36  (0.89) | 3.25  (0.51) |  | 3.23  (0.32) | 3.09  (0.28) |
| *Left-side dominant* |  | 2.99  (0.22) | 3.14  (0.39) |  | 3.07  (0.24) | 3.03  (0.33) |  | 2.98  (0.42) | 2.97  (0.16) |
|  |  |  |  |  |  |  |  |  |  |
| DFA - overall (s) |  |  |  |  |  |  |  |  |  |
| *Right-side dominant* |  | 0.69  (0.15) | 0.69  (0.13) |  | 0.70  (0.15) | 0.70  (0.14) |  | 0.71  (0.12) | 0.71  (0.17) |
| *Left-side dominant* |  | 0.74  (0.10) | 0.75  (0.12) |  | 0.70  (0.08) | 0.70  (0.07) |  | 0.70  (0.08) | 0.74  (0.10) |
|  |  |  |  |  |  |  |  |  |  |
| DFA – alpha1 (s) |  |  |  |  |  |  |  |  |  |
| *Right-side dominant* |  | 0.69  (0.11) | 0.69  (0.12) |  | 0.67  (0.14) | 0.66  (0.12) |  | 0.68  (0.12) | 0.68  (0.17) |
| *Left-side dominant* |  | 0.73  (0.11) | 0.69  (0.11) |  | 0.64  (0.10) | 0.64  (0.07) |  | 0.67  (0.07) | 0.64  (0.06) |
|  |  |  |  |  |  |  |  |  |  |
| DFA – alpha2 (s) * |  |  |  |  |  |  |  |  |  |
| *Right-side dominant* |  | 0.65  (0.28) | 0.70  (0.43) |  | 0.76  (0.34) | 0.78  (0.24) |  | 0.76  (0.24) | 0.70  (0.33) |
| *Left-side dominant* |  | 0.89  (0.35) | 0.95  (0.29) |  | 0.80  (0.23) | 0.86  (0.31) |  | 0.73  (0.17) | 0.90  (0.24) |
| Values are mean (SD) of each variable.  D: dominant leg, DFA: detrended fluctuation analysis; IQR: interquartile range; ND: non-dominant leg; Q1: first quarter; Q3: third quarter; RMS: root mean square; SD: standard deviation  * indicates differences between the left and right-side dominant participants | | | | | | | | | |

| **Supplementary Table 6:** Kinematic data of step length | | | | | | | | | |
| --- | --- | --- | --- | --- | --- | --- | --- | --- | --- |
|  |  | **Clear glass** | |  | **Left half-field eye patching** | |  | **Right half-field eye patching** | |
|  |  | *D* | *ND* |  | *D* | *ND* |  | *D* | *ND* |
| Mean (cm) |  |  |  |  |  |  |  |  |  |
| *Right-side dominant* |  | 56.30  (8.47) | 56.41  (8.23) |  | 56.08  (8.20) | 55.92  (8.05) |  | 56.05  (8.56) | 55.99  (8.52) |
| *Left-side dominant* |  | 56.17  (6.09) | 57.06  (5.97) |  | 56.32  (5.79) | 57.28  (6.04) |  | 57.03  (5.81) | 57.18  (6.16) |
|  |  |  |  |  |  |  |  |  |  |
| Min (cm) |  |  |  |  |  |  |  |  |  |
| *Right-side dominant* |  | 51.88  (8.10) | 52.44  (8.53) |  | 51.41  (8.13) | 50.99  (7.70) |  | 51.74  (8.58) | 51.89  (9.24) |
| *Left-side dominant* |  | 52.57  (6.44) | 52.89  (6.21) |  | 52.13  (5.48) | 53.47  (5.83) |  | 52.31  (7.15) | 52.26  (6.64) |
|  |  |  |  |  |  |  |  |  |  |
| Max (cm) |  |  |  |  |  |  |  |  |  |
| *Right-side dominant* |  | 59.64  (8.21) | 59.94  (8.34) |  | 59.68  (8.17) | 59.57  (8.02) |  | 59.68  (7.99) | 59.71  (8.41) |
| *Left-side dominant* |  | 60.12  (5.86) | 60.99  (6.16) |  | 60.13  (6.02) | 61.10  (6.13) |  | 60.70  (6.30) | 61.04  (6.31) |
|  |  |  |  |  |  |  |  |  |  |
| SD (cm) |  |  |  |  |  |  |  |  |  |
| *Right-side dominant* |  | 1.25  (0.27) | 1.27  (0.31) |  | 1.25  (0.28) | 1.36  (0.37) |  | 1.27  (0.28) | 1.29  (0.31) |
| *Left-side dominant* |  | 1.28  (0.32) | 1.37  (0.39) |  | 1.27  (0.31) | 1.30  (0.31) |  | 1.34  (0.32) | 1.36  (0.37) |
|  |  |  |  |  |  |  |  |  |  |
| RMS (cm) |  |  |  |  |  |  |  |  |  |
| *Right-side dominant* |  | 56.31  (8.47) | 56.43  (8.23) |  | 56.10  (8.20) | 55.94  (8.05) |  | 56.06  (8.55) | 56.01  (8.52) |
| *Left-side dominant* |  | 56.19  (6.09) | 57.07  (5.96) |  | 56.34  (5.79) | 57.30  (6.04) |  | 57.05  (5.81) | 57.20  (6.16) |
|  |  |  |  |  |  |  |  |  |  |
| Median (cm) |  |  |  |  |  |  |  |  |  |
| *Right-side dominant* |  | 56.34  (8.47) | 56.43  (8.21) |  | 56.09  (8.21) | 55.94  (8.08) |  | 56.07  (8.56) | 56.02  (8.56) |
| *Left-side dominant* |  | 56.22  (6.08) | 57.04  (5.88) |  | 56.35  (5.76) | 57.33  (6.02) |  | 57.09  (5.78) | 57.23  (6.14) |
|  |  |  |  |  |  |  |  |  |  |
| Q1 (cm) |  |  |  |  |  |  |  |  |  |
| *Right-side dominant* |  | 55.51  (8.55) | 55.61  (8.24) |  | 55.31  (8.26) | 55.06  (8.11) |  | 55.24  (8.63) | 55.15  (8.59) |
| *Left-side dominant* |  | 55.34  (6.11) | 56.15  (5.93) |  | 55.53  (5.75) | 56.44  (6.00) |  | 56.21  (5.80) | 56.33  (6.22) |
|  |  |  |  |  |  |  |  |  |  |
| Q3 (cm) |  |  |  |  |  |  |  |  |  |
| *Right-side dominant* |  | 57.14  (8.40) | 57.26  (8.20) |  | 56.90  (8.12) | 56.84  (8.06) |  | 56.90  (8.50) | 56.85  (8.45) |
| *Left-side dominant* |  | 56.98  (8.40) | 57.95  (5.98) |  | 57.16  (5.85) | 58.16  (6.08) |  | 57.91  (5.77) | 58.09  (6.11) |
|  |  |  |  |  |  |  |  |  |  |
| IQR (cm) |  |  |  |  |  |  |  |  |  |
| *Right-side dominant* |  | 1.63  (0.39) | 1.64  (0.35) |  | 1.59  (0.36) | 1.78  (0.38) |  | 1.66  (0.41) | 1.70  (0.42) |
| *Left-side dominant* |  | 1.64  (0.37) | 1.79  (0.46) |  | 1.64  (0.46) | 1.72  (0.39) |  | 1.70  (0.45) | 1.76  (0.59) |
|  |  |  |  |  |  |  |  |  |  |
| Mode (cm) |  |  |  |  |  |  |  |  |  |
| *Right-side dominant* |  | 51.88  (8.10) | 52.44  (8.53) |  | 51.41  (8.13) | 50.99  (7.70) |  | 51.74  (8.58) | 51.89  (9.24) |
| *Left-side dominant* |  | 52.57  (6.44) | 52.89  (6.21) |  | 52.13  (5.48) | 53.47  (5.83) |  | 52.31  (7.15) | 52.26  (6.64) |
|  |  |  |  |  |  |  |  |  |  |
| Skewness (cm) |  |  |  |  |  |  |  |  |  |
| *Right-side dominant* |  | -0.42  (0.87) | -0.19  (0.20) |  | -0.32  (0.80) | -0.28  (0.71) |  | -0.20  (0.27) | -0.14  (0.31) |
| *Left-side dominant* |  | -0.04  (0.22) | -0.05  (0.34) |  | -0.16  (0.23) | -0.11  (0.11) |  | -0.30  (0.47) | -0.26  (0.38) |
|  |  |  |  |  |  |  |  |  |  |
| Kurtosis (cm) |  |  |  |  |  |  |  |  |  |
| *Right-side dominant* |  | 5.32  (8.42) | 3.27  (0.36) |  | 5.10  (6.84) | 4.24  (3.96) |  | 3.62  (0.70) | 3.32  (0.48) |
| *Left-side dominant* |  | 3.32  (0.64) | 3.29  (0.58) |  | 3.58  (0.49) | 3.16  (0.27) |  | 4.14  (2.83) | 3.90  (1.39) |
|  |  |  |  |  |  |  |  |  |  |
| DFA - overall (cm) |  |  |  |  |  |  |  |  |  |
| *Right-side dominant* |  | 0.71  (0.10) | 0.70  (0.10) |  | 0.68  (0.09) | 0.70  (0.11) |  | 0.70  (0.08) | 0.71  (0.10) |
| *Left-side dominant* |  | 0.74  (0.08) | 0.76  (0.14) |  | 0.74  (0.05) | 0.71  (0.09) |  | 0.74  (0.09) | 0.74  (0.10) |
|  |  |  |  |  |  |  |  |  |  |
| DFA – alpha1 (cm) |  |  |  |  |  |  |  |  |  |
| *Right-side dominant* |  | 0.68  (0.14) | 0.68  (0.11) |  | 0.67  (0.09) | 0.64  (0.09) |  | 0.69  (0.09) | 0.68  (0.10) |
| *Left-side dominant* |  | 0.69  (0.12) | 0.70  (0.11) |  | 0.66  (0.04) | 0.71  (0.14) |  | 0.65  (0.08) | 0.64  (0.11) |
|  |  |  |  |  |  |  |  |  |  |
| DFA – alpha2 (cm) |  |  |  |  |  |  |  |  |  |
| *Right-side dominant* |  | 0.69  (0.23) | 0.74  (0.28) |  | 0.79  (0.29) | 0.81  (0.35) |  | 0.83  (0.27) | 0.77  (0.29) |
| *Left-side dominant* |  | 0.85  (0.26) | 0.87  (0.36) |  | 0.92  (0.29) | 0.81  (0.34) |  | 0.85  (0.34) | 0.99  (0.26) |
| Values are mean (SD) of each variable.  D: dominant leg, DFA: detrended fluctuation analysis; IQR: interquartile range; ND: non-dominant leg; Q1: first quarter; Q3: third quarter; RMS: root mean square; SD: standard deviation | | | | | | | | | |

| **Supplementary Table 7:** Kinematic data of step width | | | | | | | | | |
| --- | --- | --- | --- | --- | --- | --- | --- | --- | --- |
|  |  | **Clear glass** | |  | **Left half-field eye patching** | |  | **Right half-field eye patching** | |
|  |  | *D* | *ND* |  | *D* | *ND* |  | *D* | *ND* |
| Mean (cm) * |  |  |  |  |  |  |  |  |  |
| *Right-side dominant* |  | 8.86  (2.95) | 6.40  (2.94) |  | 9.03  (2.83) | 6.55  (2.87) |  | 9.27  (2.68) | 6.76  (2.81) |
| *Left-side dominant* |  | 6.13  (3.08) | 7.38  (2.17) |  | 6.15  (3.25) | 7.44  (2.31) |  | 6.11  (2.79) | 7.47  (1.96) |
|  |  |  |  |  |  |  |  |  |  |
| Min (cm) * |  |  |  |  |  |  |  |  |  |
| *Right-side dominant* |  | 4.32  (2.52) | 2.32  (2.12) |  | 4.30  (2.47) | 2.25  (1.90) |  | 4.30  (2.67) | 2.34  (2.25) |
| *Left-side dominant* |  | 1.37  (1.69) | 2.11  (1.67) |  | 0.83  (1.36) | 1.62  (1.56) |  | 1.17  (1.75) | 2.02  (2.03) |
|  |  |  |  |  |  |  |  |  |  |
| Max (cm) |  |  |  |  |  |  |  |  |  |
| *Right-side dominant* |  | 14.01  (4.25) | 11.69  (3.75) |  | 14.29  (3.20) | 12.11  (3.43) |  | 14.64  (3.39) | 12.44  (3.40) |
| *Left-side dominant* |  | 12.80  (5.37) | 13.50  (4.47) |  | 13.07  (4.55) | 13.93  (4.55) |  | 12.40  (4.33) | 14.38  (4.39) |
|  |  |  |  |  |  |  |  |  |  |
| SD (cm) * |  |  |  |  |  |  |  |  |  |
| *Right-side dominant* |  | 1.66  (0.51) | 1.64  (0.58) |  | 1.72  (0.47) | 1.70  (0.42) |  | 1.75  (0.46) | 1.71  (0.49) |
| *Left-side dominant* |  | 1.98  (0.86) | 1.96  (0.91) |  | 2.03  (0.81) | 2.02  (0.91) |  | 2.05  (0.75) | 2.07  (0.90) |
|  |  |  |  |  |  |  |  |  |  |
| RMS (cm) |  |  |  |  |  |  |  |  |  |
| *Right-side dominant* |  | 9.04  (2.91) | 6.66  (2.87) |  | 9.21  (2.78) | 6.81  (2.78) |  | 9.45  (2.65) | 7.02  (2.73) |
| *Left-side dominant* |  | 6.49  (3.09) | 7.66  (2.28) |  | 6.52  (3.24) | 7.73  (2.41) |  | 6.48  (2.80) | 7.78  (2.06) |
|  |  |  |  |  |  |  |  |  |  |
| Median (cm) * |  |  |  |  |  |  |  |  |  |
| *Right-side dominant* |  | 8.83  (2.98) | 6.34  (3.01) |  | 9.03  (2.82) | 6.46  (2.92) |  | 9.21  (2.65) | 6.74  (2.84) |
| *Left-side dominant* |  | 6.14  (3.12) | 7.36  (2.18) |  | 6.07  (3.28) | 7.46  (2.38) |  | 6.04  (2.79) | 7.47  (2.02) |
|  |  |  |  |  |  |  |  |  |  |
| Q1 (cm) * |  |  |  |  |  |  |  |  |  |
| *Right-side dominant* |  | 7.76  (2.88) | 5.29  (2.85) |  | 7.87  (2.76) | 5.42  (2.81) |  | 8.12  (2.58) | 5.57  (2.77) |
| *Left-side dominant* |  | 4.77  (2.81) | 6.16  (1.84) |  | 4.79  (3.04) | 6.16  (1.96) |  | 4.70  (2.63) | 6.10  (1.62) |
|  |  |  |  |  |  |  |  |  |  |
| Q3 (cm) |  |  |  |  |  |  |  |  |  |
| *Right-side dominant* |  | 9.92  (3.08) | 7.46  (3.14) |  | 10.16  (2.95) | 7.63  (3.05) |  | 10.37  (2.80) | 7.88  (2.97) |
| *Left-side dominant* |  | 7.32  (3.45) | 8.65  (2.69) |  | 7.46  (3.64) | 8.69  (2.73) |  | 7.40  (3.09) | 8.82  (2.36) |
|  |  |  |  |  |  |  |  |  |  |
| IQR (cm) * |  |  |  |  |  |  |  |  |  |
| *Right-side dominant* |  | 2.17  (0.67) | 2.17  (0.70) |  | 2.29  (0.63) | 2.21  (0.51) |  | 2.25  (0.62) | 2.31  (0.67) |
| *Left-side dominant* |  | 2.55  (1.09) | 2.49  (1.19) |  | 2.67  (1.27) | 2.52  (1.12) |  | 2.69  (1.08) | 2.72  (1.24) |
|  |  |  |  |  |  |  |  |  |  |
| Mode (cm) * |  |  |  |  |  |  |  |  |  |
| *Right-side dominant* |  | 4.32  (2.52) | 2.32  (2.12) |  | 4.30  (2.47) | 2.25  (1.90) |  | 4.30  (2.67) | 2.34  (2.25) |
| *Left-side dominant* |  | 1.37  (1.69) | 2.11  (1.67) |  | 0.83  (1.36) | 1.62  (1.56) |  | 1.17  (1.75) | 2.02  (2.03) |
|  |  |  |  |  |  |  |  |  |  |
| Skewness (cm) |  |  |  |  |  |  |  |  |  |
| *Right-side dominant* |  | 0.11  (0.20) | 0.27  (0.45) |  | 0.07  (0.22) | 0.30  (0.42) |  | 0.15  (0.24) | 0.19  (0.35) |
| *Left-side dominant* |  | 0.29  (0.33) | 0.10  (0.37) |  | 0.30  (0.35) | 0.06  (0.36) |  | 0.18  (0.23) | 0.11  (0.19) |
|  |  |  |  |  |  |  |  |  |  |
| Kurtosis (cm) |  |  |  |  |  |  |  |  |  |
| *Right-side dominant* |  | 3.17  (0.40) | 3.35  (0.85) |  | 3.14  (0.37) | 3.44  (0.54) |  | 3.29  (0.51) | 3.30  (0.66) |
| *Left-side dominant* |  | 3.46  (0.54) | 3.47  (0.46) |  | 3.64  (0.73) | 3.62  (0.80) |  | 3.11  (0.43) | 3.40  (0.68) |
|  |  |  |  |  |  |  |  |  |  |
| DFA - overall (cm) |  |  |  |  |  |  |  |  |  |
| *Right-side dominant* |  | 0.62  (0.10) | 0.60  (0.10) |  | 0.64  (0.13) | 0.63  (0.13) |  | 0.61  (0.17) | 0.62  (0.17) |
| *Left-side dominant* |  | 0.63  (0.12) | 0.64  (0.13) |  | 0.61  (0.08) | 0.63  (0.08) |  | 0.66  (0.11) | 0.68  (0.12) |
|  |  |  |  |  |  |  |  |  |  |
| DFA – alpha1 (cm) |  |  |  |  |  |  |  |  |  |
| *Right-side dominant* |  | 0.61  (0.10) | 0.63  (0.13) |  | 0.63  (0.14) | 0.62  (0.15) |  | 0.59  (0.12) | 0.59  (0.10) |
| *Left-side dominant* |  | 0.59  (0.05) | 0.64  (0.06) |  | 0.58  (0.09) | 0.65  (0.12) |  | 0.65  (0.11) | 0.67  (0.10) |
|  |  |  |  |  |  |  |  |  |  |
| DFA – alpha2 (cm) |  |  |  |  |  |  |  |  |  |
| *Right-side dominant* |  | 0.59  (0.17) | 0.57  (0.24) |  | 0.66  (0.18) | 0.61  (0.17) |  | 0.63  (0.45) | 0.68  (0.41) |
| *Left-side dominant* |  | 0.71  (0.19) | 0.66  (0.22) |  | 0.56  (0.22) | 0.58  (0.20) |  | 0.75  (0.19) | 0.73  (0.18) |
| Values are mean (SD) of each variable.  D: dominant leg, DFA: detrended fluctuation analysis; IQR: interquartile range; ND: non-dominant leg; Q1: first quarter; Q3: third quarter; RMS: root mean square; SD: standard deviation  * indicates differences between the left and right-side dominant participants | | | | | | | | | |

| **Supplementary Table 8:** Kinematic data of minimum toe clearance | | | | | | | | | |
| --- | --- | --- | --- | --- | --- | --- | --- | --- | --- |
|  |  | **Clear glass** | |  | **Left half-field eye patching** | |  | **Right half-field eye patching** | |
|  |  | *D* | *ND* |  | *D* | *ND* |  | *D* | *ND* |
| Mean (cm) |  |  |  |  |  |  |  |  |  |
| *Right-side dominant* |  | 1.91  (0.59) | 1.80  (0.44) |  | 1.95  (0.60) | 1.83  (0.50) |  | 1.94  (0.59) | 1.81  (0.47) |
| *Left-side dominant* |  | 1.83  (0.61) | 1.91  (0.35) |  | 1.77  (0.46) | 1.89  (0.29) |  | 1.73  (0.36) | 1.88  (0.30) |
|  |  |  |  |  |  |  |  |  |  |
| Min (cm) |  |  |  |  |  |  |  |  |  |
| *Right-side dominant* |  | 1.24  (0.57) | 1.08  (0.48) |  | 1.16  (0.63) | 1.11  (0.50) |  | 1.24  (0.62) | 1.09  (0.52) |
| *Left-side dominant* |  | 1.16  (0.56) | 1.23  (0.35) |  | 1.08  (0.48) | 1.29  (0.24) |  | 1.09  (0.38) | 1.27  (0.30) |
|  |  |  |  |  |  |  |  |  |  |
| Max (cm) |  |  |  |  |  |  |  |  |  |
| *Right-side dominant* |  | 2.64  (0.68) | 2.63  (0.57) |  | 2.74  (0.69) | 2.77  (0.77) |  | 2.75  (0.70) | 2.55  (0.57) |
| *Left-side dominant* |  | 2.48  (0.74) | 2.50  (0.44) |  | 2.43  (0.58) | 2.49  (0.43) |  | 2.43  (0.57) | 2.52  (0.48) |
|  |  |  |  |  |  |  |  |  |  |
| SD (cm) * |  |  |  |  |  |  |  |  |  |
| *Right-side dominant* |  | 0.24  (0.05) | 0.25  (0.05) |  | 0.25  (0.05) | 0.26  (0.07) |  | 0.24  (0.04) | 0.24  (0.05) |
| *Left-side dominant* |  | 0.22  (0.06) | 0.21  (0.05) |  | 0.22  (0.06) | 0.22  (0.05) |  | 0.22  (0.06) | 0.21  (0.04) |
|  |  |  |  |  |  |  |  |  |  |
| RMS (cm) |  |  |  |  |  |  |  |  |  |
| *Right-side dominant* |  | 1.93  (0.59) | 1.82  (0.44) |  | 1.97  (0.60) | 1.85  (0.50) |  | 1.96  (0.58) | 1.82  (0.47) |
| *Left-side dominant* |  | 1.85  (0.61) | 1.92  (0.35) |  | 1.79  (0.46) | 1.90  (0.29) |  | 1.74  (0.36) | 1.90  (0.30) |
|  |  |  |  |  |  |  |  |  |  |
| Median (cm) |  |  |  |  |  |  |  |  |  |
| *Right-side dominant* |  | 1.91  (0.58) | 1.79  (0.43) |  | 1.95  (0.61) | 1.82  (0.48) |  | 1.94  (0.58) | 1.80  (0.47) |
| *Left-side dominant* |  | 1.83  (0.61) | 1.91  (0.35) |  | 1.77  (0.46) | 1.89  (0.28) |  | 1.72  (0.36) | 1.88  (0.30) |
|  |  |  |  |  |  |  |  |  |  |
| Q1 (cm) |  |  |  |  |  |  |  |  |  |
| *Right-side dominant* |  | 1.76  (0.57) | 1.63  (0.42) |  | 1.79  (0.58) | 1.66  (0.48) |  | 1.79  (0.58) | 1.64  (0.45) |
| *Left-side dominant* |  | 1.69  (0.58) | 1.77  (0.32) |  | 1.63  (0.43) | 1.75  (0.26) |  | 1.59  (0.35) | 1.74  (0.29) |
|  |  |  |  |  |  |  |  |  |  |
| Q3 (cm) |  |  |  |  |  |  |  |  |  |
| *Right-side dominant* |  | 2.07  (0.60) | 1.96  (0.44) |  | 2.11  (0.63) | 2.00  (0.51) |  | 2.10  (0.60) | 1.97  (0.49) |
| *Left-side dominant* |  | 1.97  (0.64) | 2.06  (0.38) |  | 1.92  (0.50) | 2.03  (0.31) |  | 1.87  (0.38) | 2.02  (0.31) |
|  |  |  |  |  |  |  |  |  |  |
| IQR (cm) * |  |  |  |  |  |  |  |  |  |
| *Right-side dominant* |  | 0.31  (0.06) | 0.33  (0.07) |  | 0.32  (0.08) | 0.34  (0.09) |  | 0.31  (0.05) | 0.33  (0.08) |
| *Left-side dominant* |  | 0.29  (0.07) | 0.29  (0.07) |  | 0.28  (0.09) | 0.28  (0.07) |  | 0.28  (0.09) | 0.27  (0.04) |
|  |  |  |  |  |  |  |  |  |  |
| Mode (cm) |  |  |  |  |  |  |  |  |  |
| *Right-side dominant* |  | 1.24  (0.57) | 1.08  (0.48) |  | 1.16  (0.63) | 1.11  (0.50) |  | 1.24  (0.62) | 1.09  (0.52) |
| *Left-side dominant* |  | 1.16  (0.56) | 1.23  (0.35) |  | 1.08  (0.48) | 1.29  (0.24) |  | 1.09  (0.38) | 1.27  (0.30) |
|  |  |  |  |  |  |  |  |  |  |
| Skewness (cm) * |  |  |  |  |  |  |  |  |  |
| *Right-side dominant* |  | 0.06  (0.32) | 0.28  (0.30) |  | 0.05  (0.38) | 0.28  (0.50) |  | 0.11  (0.39) | 0.09  (0.30) |
| *Left-side dominant* |  | -0.05  (0.25) | -0.05  (0.33) |  | -0.04  (0.39) | -0.05  (0.36) |  | 0.10  (0.55) | 0.09  (0.35) |
|  |  |  |  |  |  |  |  |  |  |
| Kurtosis (cm) |  |  |  |  |  |  |  |  |  |
| *Right-side dominant* |  | 3.31  (0.38) | 3.59  (0.47) |  | 3.74  (0.99) | 3.93  (1.64) |  | 3.61  (0.93) | 3.22  (0.45) |
| *Left-side dominant* |  | 3.29  (0.42) | 3.27  (0.60) |  | 3.53  (0.81) | 3.03  (0.66) |  | 3.76  (1.32) | 3.38  (0.83) |
|  |  |  |  |  |  |  |  |  |  |
| DFA - overall (cm) |  |  |  |  |  |  |  |  |  |
| *Right-side dominant* |  | 0.68  (0.12) | 0.71  (0.17) |  | 0.65  (0.10) | 0.69  (0.10) |  | 0.68  (0.10) | 0.69  (0.13) |
| *Left-side dominant* |  | 0.70  (0.10) | 0.70  (0.09) |  | 0.65  (0.11) | 0.67  (0.07) |  | 0.71  (0.09) | 0.70  (0.09) |
|  |  |  |  |  |  |  |  |  |  |
| DFA – alpha1 (cm) * |  |  |  |  |  |  |  |  |  |
| *Right-side dominant* |  | 0.64  (0.10) | 0.67  (0.07) |  | 0.66  (0.11) | 0.68  (0.13) |  | 0.65  (0.11) | 0.65  (0.09) |
| *Left-side dominant* |  | 0.71  (0.11) | 0.66  (0.06) |  | 0.70  (0.07) | 0.69  (0.11) |  | 0.71  (0.08) | 0.69  (0.09) |
|  |  |  |  |  |  |  |  |  |  |
| DFA – alpha2 (cm) |  |  |  |  |  |  |  |  |  |
| *Right-side dominant* |  | 0.79  (0.24) | 0.77  (0.34) |  | 0.69  (0.28) | 0.69  (0.28) |  | 0.75  (0.20) | 0.81  (0.32) |
| *Left-side dominant* |  | 0.75  (0.25) | 0.75  (0.29) |  | 0.63  (0.29) | 0.65  (0.28) |  | 0.85  (0.26) | 0.75  (0.29) |
| Values are mean (SD) of each variable.  D: dominant leg, DFA: detrended fluctuation analysis; IQR: interquartile range; ND: non-dominant leg; Q1: first quarter; Q3: third quarter; RMS: root mean square; SD: standard deviation  * indicates differences between the left and right-side dominant participants | | | | | | | | | |

| **Supplementary Table 9:** Kinematic data of horizontal velocity at minimum toe clearance | | | | | | | | | |
| --- | --- | --- | --- | --- | --- | --- | --- | --- | --- |
|  |  | **Clear glass** | |  | **Left half-field eye patching** | |  | **Right half-field eye patching** | |
|  |  | *D* | *ND* |  | *D* | *ND* |  | *D* | *ND* |
| Mean (m/s) |  |  |  |  |  |  |  |  |  |
| *Right-side dominant* |  | 2.62  (0.50) | 2.64  (0.48) |  | 2.63  (0.50) | 2.64  (0.48) |  | 2.62  (0.50) | 2.63  (0.47) |
| *Left-side dominant* |  | 2.67  (0.29) | 2.66  (0.27) |  | 2.68  (0.27) | 2.66  (0.28) |  | 2.69  (0.28) | 2.65  (0.29) |
|  |  |  |  |  |  |  |  |  |  |
| Min (m/s) |  |  |  |  |  |  |  |  |  |
| *Right-side dominant* |  | 2.37  (0.53) | 2.38  (0.51) |  | 2.36  (0.50) | 2.38  (0.50) |  | 2.36  (0.53) | 2.38  (0.50) |
| *Left-side dominant* |  | 2.43  (0.34) | 2.35  (0.36) |  | 2.41  (0.30) | 2.38  (0.28) |  | 2.38  (0.32) | 2.41  (0.27) |
|  |  |  |  |  |  |  |  |  |  |
| Max (m/s) |  |  |  |  |  |  |  |  |  |
| *Right-side dominant* |  | 2.84  (0.49) | 2.85  (0.46) |  | 2.87  (0.46) | 2.88  (0.46) |  | 2.84  (0.46) | 2.86  (0.44) |
| *Left-side dominant* |  | 2.91  (0.27) | 2.89  (0.24) |  | 2.90  (0.27) | 2.90  (0.31) |  | 2.89  (0.26) | 2.88  (0.28) |
|  |  |  |  |  |  |  |  |  |  |
| SD (m/s) |  |  |  |  |  |  |  |  |  |
| *Right-side dominant* |  | 0.08  (0.01) | 0.08  (0.01) |  | 0.08  (0.01) | 0.08  (0.02) |  | 0.08  (0.02) | 0.08  (0.01) |
| *Left-side dominant* |  | 0.08  (0.03) | 0.08  (0.02) |  | 0.08  (0.03) | 0.08  (0.02) |  | 0.08  (0.02) | 0.08  (0.01) |
|  |  |  |  |  |  |  |  |  |  |
| RMS (m/s) |  |  |  |  |  |  |  |  |  |
| *Right-side dominant* |  | 2.62  (0.50) | 2.64  (0.48) |  | 2.63  (0.50) | 2.64  (0.48) |  | 2.62  (0.50) | 2.64  (0.47) |
| *Left-side dominant* |  | 2.67  (0.29) | 2.66  (0.27) |  | 2.68  (0.27) | 2.66  (0.28) |  | 2.69  (0.28) | 2.65  (0.29) |
|  |  |  |  |  |  |  |  |  |  |
| Median (m/s) |  |  |  |  |  |  |  |  |  |
| *Right-side dominant* |  | 2.62  (0.50) | 2.64  (0.48) |  | 2.63  (0.50) | 2.64  (0.48) |  | 2.62  (0.50) | 2.64  (0.47) |
| *Left-side dominant* |  | 2.67  (0.29) | 2.66  (0.27) |  | 2.68  (0.27) | 2.66  (0.28) |  | 2.69  (0.28) | 2.65  (0.29) |
|  |  |  |  |  |  |  |  |  |  |
| Q1 (m/s) |  |  |  |  |  |  |  |  |  |
| *Right-side dominant* |  | 2.57  (0.51) | 2.59  (0.49) |  | 2.58  (0.50) | 2.59  (0.49) |  | 2.57  (0.50) | 2.58  (0.47) |
| *Left-side dominant* |  | 2.62  (0.30) | 2.60  (0.27) |  | 2.62  (0.28) | 2.61  (0.27) |  | 2.64  (0.28) | 2.59  (0.29) |
|  |  |  |  |  |  |  |  |  |  |
| Q3 (m/s) |  |  |  |  |  |  |  |  |  |
| *Right-side dominant* |  | 2.67  (0.50) | 2.69  (0.47) |  | 2.68  (0.49) | 2.69  (0.47) |  | 2.68  (0.49) | 2.69  (0.46) |
| *Left-side dominant* |  | 2.72  (0.29) | 2.71  (0.27) |  | 2.73  (0.27) | 2.71  (0.28) |  | 2.74  (0.28) | 2.70  (0.29) |
|  |  |  |  |  |  |  |  |  |  |
| IQR (m/s) |  |  |  |  |  |  |  |  |  |
| *Right-side dominant* |  | 0.10  (0.02) | 0.10  (0.02) |  | 0.10  (0.02) | 0.11  (0.02) |  | 0.11  (0.02) | 0.10  (0.02) |
| *Left-side dominant* |  | 0.11  (0.04) | 0.10  (0.02) |  | 0.10  (0.04) | 0.10  (0.03) |  | 0.10  (0.02) | 0.11  (0.02) |
|  |  |  |  |  |  |  |  |  |  |
| Mode (m/s) |  |  |  |  |  |  |  |  |  |
| *Right-side dominant* |  | 2.37  (0.53) | 2.38  (0.51) |  | 2.36  (0.50) | 2.38  (0.50) |  | 2.36  (0.53) | 2.38  (0.50) |
| *Left-side dominant* |  | 2.43  (0.34) | 2.35  (0.36) |  | 2.41  (0.30) | 2.38  (0.28) |  | 2.38  (0.32) | 2.41  (0.27) |
|  |  |  |  |  |  |  |  |  |  |
| Skewness (m/s) |  |  |  |  |  |  |  |  |  |
| *Right-side dominant* |  | -0.14  (0.33) | -0.18  (0.39) |  | -0.11  (0.28) | -0.09  (0.36) |  | -0.13  (0.25) | -0.11  (0.27) |
| *Left-side dominant* |  | -0.09  (0.28) | -0.29  (1.04) |  | -0.17  (0.18) | -0.13  (0.21) |  | -0.37  (0.51) | -0.07  (0.27) |
|  |  |  |  |  |  |  |  |  |  |
| Kurtosis (m/s) |  |  |  |  |  |  |  |  |  |
| *Right-side dominant* |  | 3.32  (0.51) | 3.49  (0.97) |  | 3.56  (0.69) | 3.62  (0.97) |  | 3.27  (0.47) | 3.39  (0.53) |
| *Left-side dominant* |  | 3.24  (0.36) | 5.74  (7.24) |  | 3.44  (0.46) | 3.53  (0.64) |  | 4.10  (1.49) | 3.24  (0.46) |
|  |  |  |  |  |  |  |  |  |  |
| DFA - overall (m/s) |  |  |  |  |  |  |  |  |  |
| *Right-side dominant* |  | 0.63  (0.10) | 0.63  (0.10) |  | 0.60  (0.08) | 0.62  (0.07) |  | 0.64  (0.12) | 0.61  (0.11) |
| *Left-side dominant* |  | 0.61  (0.09) | 0.59  (0.08) |  | 0.61  (0.07) | 0.58  (0.10) |  | 0.63  (0.10) | 0.62  (0.12) |
|  |  |  |  |  |  |  |  |  |  |
| DFA – alpha1 (m/s) |  |  |  |  |  |  |  |  |  |
| *Right-side dominant* |  | 0.62  (0.09) | 0.61  (0.11) |  | 0.61  (0.09) | 0.60  (0.08) |  | 0.61  (0.08) | 0.57  (0.10) |
| *Left-side dominant* |  | 0.66  (0.09) | 0.63  (0.08) |  | 0.62  (0.06) | 0.60  (0.10) |  | 0.61  (0.08) | 0.68  (0.08) |
|  |  |  |  |  |  |  |  |  |  |
| DFA – alpha2 (m/s) |  |  |  |  |  |  |  |  |  |
| *Right-side dominant* |  | 0.66  (0.21) | 0.70  (0.29) |  | 0.59  (0.19) | 0.67  (0.19) |  | 0.65  (0.19) | 0.64  (0.19) |
| *Left-side dominant* |  | 0.50  (0.30) | 0.50  (0.22) |  | 0.66  (0.16) | 0.54  (0.24) |  | 0.56  (0.26) | 0.65  (0.25) |
| Values are mean (SD) of each variable.  D: dominant leg, DFA: detrended fluctuation analysis; IQR: interquartile range; ND: non-dominant leg; Q1: first quarter; Q3: third quarter; RMS: root mean square; SD: standard deviation | | | | | | | | | |

| **Supplementary Table 10:** Kinematic data of cadence | | | | |
| --- | --- | --- | --- | --- |
|  |  | **Clear glass** | **Left half-field eye patching** | **Right half-field eye patching** |
| Cadence (steps/min) |  |  |  |  |
| *Right-side dominant* |  | 103.6  (10.4) | 104.2  (10.6) | 104.2  (10.2) |
| *Left-side dominant* |  | 102.3  (10.6) | 101.8  (10.8) | 101.2  (10.0) |
| Values are mean (SD) of each variable. | | | | |

| **Supplementary Table 11:** Hip joint kinematics | | | | | | | | | |
| --- | --- | --- | --- | --- | --- | --- | --- | --- | --- |
|  |  | **Clear glass** | |  | **Left half-field eye patching** | |  | **Right half-field eye patching** | |
|  |  | *D* | *ND* |  | *D* | *ND* |  | *D* | *ND* |
| ROM (°) |  |  |  |  |  |  |  |  |  |
| *Right-side dominant* |  | 47.2  (5.5) | 47.3  (5.7) |  | 46.8  (5.5) | 47.5  (5.9) |  | 47.0  (5.3) | 47.2  (6.0) |
| *Left-side dominant* |  | 45.9  (7.1) | 45.5  (2.8) |  | 46.0  (6.3) | 45.5  (3.2) |  | 45.9  (6.8) | 45.6  (3.2) |
|  |  |  |  |  |  |  |  |  |  |
| Average SD (°) |  |  |  |  |  |  |  |  |  |
| *Right-side dominant* |  | 1.32  (0.29) | 1.19  (0.16) |  | 1.34  (0.36) | 1.24  (0.26) |  | 1.30  (0.25) | 1.21  (0.23) |
| *Left-side dominant* |  | 1.28  (0.32) | 1.29  (0.32) |  | 1.25  (0.24) | 1.23  (0.25) |  | 1.22  (0.23) | 1.26  (0.27) |
|  |  |  |  |  |  |  |  |  |  |
| Peak flexion (°) |  |  |  |  |  |  |  |  |  |
| *Right-side dominant* |  | 33.8  (5.9) | 32.8  (5.0) |  | 33.8  (6.0) | 32.8  (4.9) |  | 33.7  (5.5) | 32.5  (4.5) |
| *Left-side dominant* |  | 33.1  (6.9) | 34.4  (6.3) |  | 33.0  (6.9) | 34.2  (6.6) |  | 33.3  (6.9) | 34.5  (6.5) |
|  |  |  |  |  |  |  |  |  |  |
| Peak extension (°) |  |  |  |  |  |  |  |  |  |
| *Right-side dominant* |  | 13.3  (6.3) | 14.5  (5.7) |  | 13.1  (6.5) | 14.7  (5.8) |  | 13.4  (6.1) | 14.6  (5.7) |
| *Left-side dominant* |  | 12.8  (8.6) | 11.1  (5.7) |  | 13.0  (8.4) | 11.3  (6.3) |  | 12.6  (8.5) | 11.1  (5.9) |
| Values are mean (SD) of each variable.  D: dominant leg, ND: non-dominant leg; ROM: range of motion; SD: standard deviation | | | | | | | | | |

| **Supplementary Table 12:** Knee joint kinematics | | | | | | | | | |
| --- | --- | --- | --- | --- | --- | --- | --- | --- | --- |
|  |  | **Clear glass** | |  | **Left half-field eye patching** | |  | **Right half-field eye patching** | |
|  |  | *D* | *ND* |  | *D* | *ND* |  | *D* | *ND* |
| ROM (°) * |  |  |  |  |  |  |  |  |  |
| *Right-side dominant* |  | 67.3  (6.6) | 65.5  (6.2) |  | 67.2  (7.0) | 65.6  (6.5) |  | 66.9  (6.5) | 65.5  (6.5) |
| *Left-side dominant* |  | 63.6  (4.4) | 64.9  (3.9) |  | 63.5  (4.2) | 64.7  (4.6) |  | 63.4  (4.4) | 64.5  (4.0) |
|  |  |  |  |  |  |  |  |  |  |
| Average SD (°) |  |  |  |  |  |  |  |  |  |
| *Right-side dominant* |  | 1.87  (0.46) | 1.72  (0.29) |  | 1.88  (0.48) | 1.76  (0.37) |  | 1.84  (0.37) | 1.73  (0.34) |
| *Left-side dominant* |  | 1.82  (0.51) | 1.86  (0.44) |  | 1.79  (0.43) | 1.80  (0.34) |  | 1.77  (0.35) | 1.86  (0.36) |
|  |  |  |  |  |  |  |  |  |  |
| Peak flexion (°) |  |  |  |  |  |  |  |  |  |
| *Right-side dominant* |  | 69.9  (5.4) | 67.9  (6.0) |  | 70.2  (5.7) | 68.2  (6.0) |  | 69.8  (5.6) | 67.7  (6.1) |
| *Left-side dominant* |  | 68.6  (4.9) | 71.2  (3.3) |  | 68.4  (4.7) | 70.7  (2.9) |  | 68.3  (5.0) | 70.7  (4.0) |
|  |  |  |  |  |  |  |  |  |  |
| Midstance min. flex. (°) * |  |  |  |  |  |  |  |  |  |
| *Right-side dominant* |  | 4.18  (5.17) | 3.22  (5.42) |  | 4.37  (5.21) | 3.20  (5.39) |  | 4.10  (4.82) | 3.04  (5.32) |
| *Left-side dominant* |  | 6.82  (4.38) | 8.64  (5.09) |  | 6.52  (4.04) | 8.56  (5.30) |  | 6.77  (3.43) | 8.78  (5.25) |
|  |  |  |  |  |  |  |  |  |  |
| Initial contact flexion (°) |  |  |  |  |  |  |  |  |  |
| *Right-side dominant* |  | 6.22  (8.68) | 5.39  (7.11) |  | 6.50  (8.35) | 5.69  (7.35) |  | 6.80  (8.90) | 5.70  (7.35) |
| *Left-side dominant* |  | 7.94  (6.12) | 9.15  (8.48) |  | 7.53  (4.92) | 8.53  (7.45) |  | 7.12  (4.93) | 8.83  (7.57) |
| Values are mean (SD) of each variable.  D: dominant leg, ND: non-dominant leg; ROM: range of motion; SD: standard deviation  * indicates differences between the left and right-side dominant participants | | | | | | | | | |

| **Supplementary Table 13:** Ankle joint kinematics | | | | | | | | | |
| --- | --- | --- | --- | --- | --- | --- | --- | --- | --- |
|  |  | **Clear glass** | |  | **Left half-field eye patching** | |  | **Right half-field eye patching** | |
|  |  | *D* | *ND* |  | *D* | *ND* |  | *D* | *ND* |
| ROM (°) * |  |  |  |  |  |  |  |  |  |
| *Right-side dominant* |  | 30.3  (5.3) | 31.3  (6.2) |  | 30.0  (5.8) | 31.0  (6.2) |  | 29.8  (5.4) | 30.8  (6.2) |
| *Left-side dominant* |  | 29.7  (5.3) | 26.6  (5.5) |  | 29.0  (4.7) | 26.6  (5.1) |  | 30.0  (5.3) | 26.5  (6.0) |
|  |  |  |  |  |  |  |  |  |  |
| Average SD (°) |  |  |  |  |  |  |  |  |  |
| *Right-side dominant* |  | 1.23  (0.20) | 1.20  (0.17) |  | 1.23  (0.19) | 1.24  (0.21) |  | 1.19  (0.17) | 1.21  (0.18) |
| *Left-side dominant* |  | 1.23  (0.24) | 1.22  (0.41) |  | 1.20  (0.24) | 1.22  (0.43) |  | 1.25  (0.34) | 1.25  (0.37) |
|  |  |  |  |  |  |  |  |  |  |
| Peak flexion (°) |  |  |  |  |  |  |  |  |  |
| *Right-side dominant* |  | 18.3  (3.6) | 17.7  (4.2) |  | 18.6  (3.5) | 17.7  (3.8) |  | 18.3  (3.6) | 17.4  (4.3) |
| *Left-side dominant* |  | 17.3  (3.6) | 19.1  (3.6) |  | 17.1  (3.1) | 19.1  (3.2) |  | 17.4  (3.3) | 19.3  (3.3) |
|  |  |  |  |  |  |  |  |  |  |
| Peak extension (°) * |  |  |  |  |  |  |  |  |  |
| *Right-side dominant* |  | 12.0  (4.0) | 13.6  (6.2) |  | 11.4  (4.3) | 13.3  (6.1) |  | 11.5  (4.1) | 13.5  (5.9) |
| *Left-side dominant* |  | 12.4  (5.4) | 7.5  (5.1) |  | 11.9  (5.0) | 7.4  (4.8) |  | 12.6  (5.2) | 7.2  (5.3) |
|  |  |  |  |  |  |  |  |  |  |
| Initial contact flexion (°) * |  |  |  |  |  |  |  |  |  |
| *Right-side dominant* |  | 6.46  (2.73) | 5.41  (2.43) |  | 6.57  (2.79) | 5.48  (2.05) |  | 6.58  (2.62) | 5.37  (2.58) |
| *Left-side dominant* |  | 3.95  (3.24) | 5.00  (3.12) |  | 3.79  (2.77) | 4.82  (3.17) |  | 3.92  (3.02) | 5.00  (3.17) |
| Values are mean (SD) of each variable.  D: dominant leg, ND: non-dominant leg; ROM: range of motion; SD: standard deviation  * indicates differences between the left and right-side dominant participants | | | | | | | | | |

| **Supplementary Table 14:** Kinematic data of average lateral pelvic translation | | | | |
| --- | --- | --- | --- | --- |
|  |  | **Clear glass** | **Left half-field eye patching** | **Right half-field eye patching** |
| Trend - linear regression slope (mm) |  |  |  |  |
| *Right-side dominant* |  | 0.00  (0.04) | 0.00  (0.05) | 0.01  (0.05) |
| *Left-side dominant* |  | -0.01  (0.06) | 0.01  (0.04) | -0.01  (0.06) |
|  |  |  |  |  |
| Trend – p-value (mm) |  |  |  |  |
| *Right-side dominant* |  | 0.21  (0.37) | 0.09  (0.16) | 0.11  (0.20) |
| *Left-side dominant* |  | 0.04  (0.08) | 0.27  (0.33) | 0.13  (0.27) |
|  |  |  |  |  |
| Mean (mm) |  |  |  |  |
| *Right-side dominant* |  | 0.14  (0.08) | 0.13  (0.05) | 0.12  (0.08) |
| *Left-side dominant* |  | 0.12  (0.10) | 0.10  (0.07) | 0.11  (0.06) |
|  |  |  |  |  |
| Median (mm) |  |  |  |  |
| *Right-side dominant* |  | -0.01  (0.83) | 0.16  (1.07) | 0.32  (0.65) |
| *Left-side dominant* |  | -0.20  (1.01) | 0.62  (1.76) | 0.26  (1.18) |
|  |  |  |  |  |
| Mode (mm) * |  |  |  |  |
| *Right-side dominant* |  | -39.2  (14.8) | -37.1  (9.4) | -36.2  (9.1) |
| *Left-side dominant* |  | -41.9  (13.4) | -49.8  (19.6) | -45.4  (8.2) |
|  |  |  |  |  |
| SD (mm) * |  |  |  |  |
| *Right-side dominant* |  | 13.0  (3.5) | 13.1  (3.2) | 13.0  (2.9) |
| *Left-side dominant* |  | 15.5  (3.7) | 15.9  (3.3) | 15.4  (2.7) |
|  |  |  |  |  |
| Skewness (mm) |  |  |  |  |
| *Right-side dominant* |  | 0.02  (0.24) | 0.03  (0.32) | -0.03  (0.30) |
| *Left-side dominant* |  | -0.01  (0.17) | -0.12  (0.50) | -0.09  (0.24) |
|  |  |  |  |  |
| Kurtosis (mm) |  |  |  |  |
| *Right-side dominant* |  | 3.39  (0.58) | 3.29  (0.32) | 3.03  (0.37) |
| *Left-side dominant* |  | 2.87  (0.45) | 3.38  (0.72) | 3.04  (0.25) |
| Values are mean (SD) of each variable.  SD: standard deviation  * indicates differences between the left and right-side dominant participants | | | | |

| **Supplementary Table 15:** Kinematic data of IQR of lateral pelvic translation | | | | |
| --- | --- | --- | --- | --- |
|  |  | **Clear glass** | **Left half-field eye patching** | **Right half-field eye patching** |
| Number of corrective cycles |  |  |  |  |
| *Right-side dominant* |  | 10.1  (1.0) | 10.1  (1.2) | 10.1  (1.1) |
| *Left-side dominant* |  | 9.7  (1.2) | 9.4  (1.4) | 9.7  (1.0) |
| Values are mean (SD) of each variable.  IQR: interquartile range | | | | |

| **Supplementary Table 16:** Kinematic data of pelvic butterfly plot | | | | | | | | | |
| --- | --- | --- | --- | --- | --- | --- | --- | --- | --- |
|  |  | **Clear glass** | |  | **Left half-field eye patching** | |  | **Right half-field eye patching** | |
|  |  | *D* | *ND* |  | *D* | *ND* |  | *D* | *ND* |
| PLD (mm) |  |  |  |  |  |  |  |  |  |
| *Right-side dominant* |  | 34.7  (8.1) | |  | 34.3  (7.9) | |  | 34.9  (8.0) | |
| *Left-side dominant* |  | 32.4  (13.3) | |  | 32.9  (13.8) | |  | 33.8  (13.7) | |
|  |  |  |  |  |  |  |  |  |  |
| Area (mm^2^) |  |  |  |  |  |  |  |  |  |
| *Right-side dominant* |  | 193.7  (77.1) | 187.6  (135.6) |  | 199.0  (85.8) | 203.9  (155.1) |  | 209.5  (69.2) | 208.7  (161.1) |
| *Left-side dominant* |  | 219.2  (233.4) | 224.3  (185.6) |  | 222.2  (230.1) | 218.1  (200.3) |  | 223.8  (249.0) | 227.0  (185.2) |
| Values are mean (SD) of each variable.  D: dominant leg, ND: non-dominant leg; PLD: pelvic lateral displacement | | | | | | | | | |
